# Definition of a sensory lexicon and development of sensory wheels of eighteen monovarietal Italian white wines

**DOI:** 10.1002/jsfa.70465

**Published:** 2026-01-28

**Authors:** Paola Piombino, Elisabetta Pittari, Roberto Salvatore Di Fede, Maria Tiziana Lisanti, Silvia Carlin, Andrea Curioni, Giovanni Luzzini, Christine Mayr Marangon, Matteo Marangon, Fulvio Mattivi, Maria Alessandra Paissoni, Giuseppina Paola Parpinello, Maurizio Piergiovanni, Arianna Ricci, Susana Río Segade, Luca Rolle, Maurizio Ugliano, Luigi Moio

**Affiliations:** ^1^ Department of Agricultural Sciences, Division of Vine and Wine Sciences University of Napoli Federico II Avellino (AV) Italy; ^2^ Center Research and Innovation Fondazione Edmund Mach San Michele all'Adige (TN) Italy; ^3^ Department of Agronomy, Food, Natural Resources, Animals and Environment (DAFNAE) University of Padova Legnaro (PD) Italy; ^4^ Department of Biotechnology University of Verona San Pietro in Cariano (VR) Italy; ^5^ Department of Agricultural, Forest and Food Sciences University of Torino Grugliasco (TO) Italy; ^6^ Department of Agricultural and Food Sciences University of Bologna Cesena (FC) Italy; ^7^ Centre Agriculture Food Environment (C3A) University of Trento San Michele all'Adige (TN) Italy; ^8^ Department of Chemistry, Life Sciences and Environmental Sustainability University of Parma Parma Italy

**Keywords:** aroma, descriptive sensory profiles, flavor vocabulary, model wheels, mouthfeel, taste

## Abstract

**BACKGROUND:**

Italy harbors one of the richest grapevine biodiversities worldwide, yet the sensory identity of wines from many native cultivars remains poorly defined despite their relevance on the market at regional, national, or international levels. This study provides a systematic sensory characterization of 18 Italian monovarietal white wines, analyzed across 246 commercial samples, including wines never investigated before by sensory analysis. Analysis of variance, hierarchical cluster analysis, and principal component analysis were applied to Rate‐All‐That‐Apply (RATA) data performed by trained panelists to define a lexicon and identify the sensory attributes characterizing and discriminating the 18 wine types.

**RESULTS:**

A statistically based lexicon comprising 29 olfactory and seven taste/mouthfeel descriptors was defined. Multivariate statistics showed that the 18 monovarietal wine types belong to four main olfactory dimensions, labeled as fruity–balsamic, thiolic–mineral, floral–sweet, and toasty–dried. A three‐dimensional space was defined along the four olfactory directions. Müller Thurgau, Gewürztraminer, Albana, and Falanghina emerged as the most representative wines in these directions, outlining the vertices of a spatial framework within and around which the other wines are distributed. Sensory wheels representing structured visual synthesis of the most relevant attributes (odor, taste, mouthfeel) were developed as ‘identity models’, providing systematic tools for defining wines' varietal typicality.

**CONCLUSION:**

Results are relevant for enologists and wine sellers as standardized reference sensory models for production and communication, as benchmarks for PDO/PGI quality control and disciplinary improvement, for researchers to advance modeling of wine sensory quality through sensometabolomic wine studies. Results also support the international recognition and valorization of Italian grapevine biodiversity according to Agenda 2030 sustainable development goals. © 2026 The Author(s). *Journal of the Science of Food and Agriculture* published by John Wiley & Sons Ltd on behalf of Society of Chemical Industry.

## INTRODUCTION

According to the latest OIV (International Organisation of Vine and Wine) report, Italy is the second‐largest wine producer globally^1^ and possesses one of the most extensive ampelographic platforms, with 644 *Vitis vinifera* L. varieties listed in the National Catalogue,^2^ including numerous white grapes. This biodiversity contributes to a wide sensory range, particularly in monovarietal wines typical of many PDO (Protected Designation of Origin) and PGI (Protected Geographical Indication) labels. Among the factors influencing sensory typicality, grape variety plays a central role.^3^ Despite Italy's rich varietal heritage and widespread monovarietal production, sensory characterization remains limited, and no specific lexicon is available.

A sensory lexicon is a standardized set of terms used to describe a product's sensory properties, translating subjective perceptions into objective descriptors.^4^, ^5^, ^6^ In wine, it includes attributes related to appearance, aroma, flavor, and mouthfeel. For a lexicon to be effective, it must encompass all relevant descriptors to distinguish between products. Various frameworks, such as odor, flavor, and mouthfeel ‘wheels’, have been developed specifically for wine.^4^, ^7^, ^8^ Tools like the Wine Aroma Wheel have enhanced communication within the wine industry, promoted consistency in sensory evaluation, and supported consumer education.^9^ Similar models could be adapted for specific wine typologies.

Lexicons also form the basis for panel training and descriptive analysis, which define sensory profiles and key dimensions. These profiles structure attributes such as aroma and taste, highlighting product distinctiveness. Techniques like descriptive analysis (DA) and multivariate statistics, including principal component analysis (PCA), are widely used to explore and visualize sensory dimensions.^10^, ^11^ These methods support wine classification, scientific understanding, winemaking decisions, and market positioning.

Recently, the Rate‐All‐That‐Apply (RATA) method has emerged as a valid alternative to DA, offering efficiency and reliability.^11^, ^12^ RATA, derived from Check‐All‐That‐Apply (CATA), allows assessors to select applicable descriptors from a predefined list and rate their intensity. It has been successfully applied to various wine types. In a study by Rabitti *et al*.,^13^ 12 semi‐trained judges evaluated 46 wines in duplicate using RATA, demonstrating good repeatability and clear differentiation among wine types.

Sensory characterization of Italian monovarietal wines is essential to understanding the organoleptic traits of each variety. While international white wines such as Chardonnay and Sauvignon Blanc are well documented,^14^ Italian wines lack comprehensive sensory profiling despite their market relevance. Some studies include sensory data,^15^, ^16^, ^17^, ^18^ but few focus on profiling single‐varietal wines from native grapes.^19^, ^20^, ^21^, ^22^ This gap limits production and marketing strategies based on sensory specificity, reducing competitiveness. International interest in Italian cultivars is growing; white varieties such as Fiano, Vermentino, Falanghina, Garganega, Greco, Inzolia, Moscato Giallo, Pecorino, Trebbiano, Verdicchio, and Verduzzo are now cultivated in Australia.^23^

This study presents the first comprehensive sensory characterization of young monovarietal Italian white wines from 18 native grape cultivars. The varieties include Lugana, Gewürztraminer, Cortese, Erbaluce, Albana, Garganega, Ribolla Gialla, Vermentino, Arneis, Falanghina, Greco di Tufo, Müller Thurgau, Pallagrello Bianco, Fiano, Nosiola, Pinot Grigio, Verdicchio, and Vernaccia. Pinot Grigio is the most cultivated, with approximately 31 000 ha,^24^ followed by Vermentino and Garganega (~9800 ha each). Nosiola and Pallagrello Bianco are niche varieties, with 79 ha and 55 ha, respectively. Most of these have never been previously investigated (e.g., Nosiola, Pallagrello Bianco, Erbaluce, Albana).

The study aims at filling a gap, given that the few available sensory data are not comparable, as they were obtained using different methodologies. Considering the interest in deepening the understanding of the sensory dimensions and descriptive lexicon specific to Italian monovarietal white wines, this study addressed the following specific objectives: (i) to develop a specific sensory lexicon based on 246 commercial labels for 18 monovarietal Italian white wines; (ii) to define the olfactory dimensions across the wine samples; (iii) to characterize, describe and discriminate the sensory patterns (taste, mouthfeel, and odor) of each monovarietal; and finally (iv) to build sensory wheels for each monovarietal wine as models of varietal sensory identity. Conducted within the framework of the D‐Wines project *‘*The aroma diversity of Italian white wines’, this study contributes to advancing the overall scientific understanding of wines from the key Italian autochthonous white grape cultivars, and the data here presented are part of a sensory–chemical multiparametric dataset on a common representative wines sample set.^25^, ^26^

## MATERIALS AND METHODS

### Wine samples

A total of 246 monovarietal white wines (vintage 2019) from 18 Italian grape cultivars were collected from nine Italian regions. For each cultivar, between 8 and 21 different commercial wines, all produced without wood refinement, were sampled from the main geographical areas of production: 21 Lugana (Veneto), 17 Gewürztraminer (Trentino Alto Adige), 16 Cortese (Piemonte), 15 Erbaluce (Piemonte), 14 Albana (Emilia‐Romagna), 14 Garganega (Veneto), 14 Ribolla Gialla (Friuli Venezia Giulia), 14 Vermentino (Sardegna), 13 Arneis (Piemonte), 13 Falanghina (Campania), 13 Greco di Tufo (Campania), 13 Müller Thurgau (Trentino Alto Adige), 13 Pallagrello Bianco (Campania), 12 Fiano (Campania), 12 Nosiola (Trentino Alto Adige), 12 Pinot Grigio (Friuli Venezia Giulia /Veneto/Trentino Alto Adige), 11 Verdicchio (Marche), 8 Vernaccia (Toscana).

The bottles were stored at cellar temperature (15 ± 2 °C) until analysis, which was performed at a young stage of development (10–15 months from the harvest date), chosen as it corresponds to the most frequently consumed stage for these styles of dry white wines.

### Chemical analyses

The basic wine parameters (lactic acid, malic acid, tartaric acid, total acidity, pH, volatile acidity, alcohol content, residual reducing sugars, and total dry extract) were determined by Fourier transform infrared spectroscopy using a commercial WineScan analyzer (FOSS A/S, Hillerød, Denmark).^27^ Non‐reducing dry extract was calculated by subtracting the residual sugars content from the total dry extract value. Free and total sulfur dioxide contents were determined using the OIV‐MA‐AS323‐04B method.^28^

### Sensory analysis

#### Panel and ethics

A total of 12 panelists (22–50 years old, five males and seven females) were recruited among students and researchers from the University of Naples Federico II (Department of Agricultural Sciences, Division of Vine and Wine Sciences). Panelists were selected based on sensory abilities, interest, and availability; all were experienced wine tasters with prior involvement in descriptive sensory analysis. All procedures complied with the ethical standards of the institutional and/or national research committee and with the 1964 Helsinki Declaration and its later amendments or comparable ethical standards. The study protocol has been approved by the Ethics Committee for Nonbiomedical Human Research (CERSUB) of the University of Naples Federico II (PG/2025/0055508). Participation was voluntary, and informed consent was obtained prior to the experiments; tasters were required to sign a form disclosing the type of research, voluntary participation, and agreement to taste and spit reference solutions and wines. Participants were also informed of their right to withdraw at any time from the study without providing any justification. All data were collected anonymously.

The panel training and the sensory assessment of wine samples were based on previous studies, with modifications detailed below.^25^, ^29^, ^30^

#### Training phase

The training phase aimed at equipping panelists with the ability to recognize, discriminate, and describe olfactory and taste/mouthfeel stimuli using a standardized approach.

##### Training on olfactory stimuli

Panelists were trained to identify 62 odor standards derived from established literature^31^, ^32^, ^33^ and non‐scientific sources (disciplinaries, consortium/producers' websites, and informative journals) to capture the descriptors most used for defining the aromatic profiles of the studied single‐varietal white wines. The standards represented 16 white wine odor families: fruity, citrus, exotic fruit, dried fruit, floral, vegetal, balsamic, spices, woody, aromatic herbs, sweet odors, undergrowth, lactic, thiolic (Sauvignon Blanc‐like profile), mineral, and off‐odors (Table 1).

**Table 1:** Reference standards used to train the assessors in recognizing and distinguishing wine odors

Family	Descriptor	Odor reference (source/brand)	Concentration or quantity
1. Fruity	Peach	peach juice (Santal)	30 mL
	Apricot	apricot juice (Santal)	30 mL
	Pear	pear juice (Santal)	30 mL
	Apple	apple juice (Santal)	30 mL
	Green apple	green apple juice (Santal)	30 mL
	Banana	homogenized banana (Mellin)	2 teaspoons
	Red fruit	red fruit juice (Santal)	30 mL
2. Citric	Lemon	lemon peel	3–4 chopped pieces
	Grapefruit	grapefruit peel	3–4 chopped pieces
	Lime	lime peel	3–4 chopped pieces
	Orange	orange peel	3–4 chopped pieces
3. Exotic fruit	Tropical fruit	exotic juice (Santal)	30 mL
	Pineapple	pineapple juice (Santal)	30 mL
	Mango	dehydrated mango	2–3 chopped units
4. Sweet odors	Honey	honey Millefiori	3 teaspoons
	Cotton candy	marshmallow	2 chopped units
	Caramel	liquid caramel (Fabbri)	3 teaspoon
5. Dried fruit	Almond	almond pastry aroma (Paneangeli)	3 drops on cotton wool
	Walnut	walnut	7 chopped units
	Hazelnut	hazelnut	5 chopped units
	Coconut	coconut powder	4 g
6. Spices	Saffron	saffron powder	0.5 g
	Ginger	ginger powder	2 g
	Cinnamon	cinnamon powder	0.5 g
	Vanilla	vanilla pastry aroma (Paneangeli)	2–3 drops on cotton wool
	Liquorice	liquorice candies (Amarelli)	10 mL of a solution made of 20 candies macerated in 200 mL water
	Cloves	cloves powder	0.5 g
	Anise	anise powder	1 g
	Fennel	fennel seeds	0.5 g
7. Floral	Floral	bar soap Dance of flowers (L'Erbolario)	2 g (grated)
	Rose	rose distilled water (Roberts)	20 mL
	Lavender	lavender essential oil	2 drops on cotton wool
	Wildflowers	wildflowers pastry aroma (Paneangeli)	3 drops on cotton wool
	Orange blossom	orange blossom pastry aroma (Paneangeli)	2 drops on cotton wool
	Orchid	orchid essential oil	5–6 drops on cotton wool
	Chamomile	chamomile teabag (Bonomelli)	30 mL of a solution made of 5 teabags soaked in 500 mL hot water
	Citronella	citronella essential oil	1–2 drops on cotton wool
8. Balsamic	Balsamic	essential oils mixture	2–3 drops on cotton wool
	Eucalyptus	eucalyptus essential oil	2–3 drops on cotton wool
	Camphor	camphor skin oil (Marco Viti)	2–3 drops on cotton wool
	Mint	mint essential oil	2–3 drops on cotton wool
9. Aromatic herbs	Sage	sage in powder	2 g
	Mediterranean herbs	natural mixes of sea salt and herbs (Ariosto)	1 g
	Rosemary	rosemary in powder	2 g
10. Undergrowth	Hearty	wet earth	10 g
	Mushroom	dried porcini mushrooms	2 chopped units
11. Vegetal	Leaf	leaf	5 g of chopped leaves
	Tomato leaf	tomato branch	1 chopped branch
	Celery	celery stalk	2 g of chopped stokes
	Grass	grass	5 g of chopped grass
	Vegetables	spinach leaf	5 g of chopped leaves
12. Woody	Woody	oak chips medium toasting	10 mL of a solution made of 5 g L^−1^ macerated oak chips for 10 days in 12% hydroalcoholic solution
13. Lactic	Butter	butter	10 g
	Cheese	grated table cheese	5 g
14. Thiolic	Sauvignon Blanc profile	Sauvignon Blanc wine (Südtirol‐Alto Adige DOC Sauvignon Lahn 2019 St Michael‐Eppan) and standard memorized from previous experiences	35 mL
	Cat urine/box tree		
15. Mineral	Flint	matchstick head	2–3 matchstick heads
	Conceptual	‘multimodal concept combining olfaction, gustation and mouthfeel sensations. The main olfactory dimensions reported were stone/flint‐related attributes, iodine, and sea‐related terms (e.g., shellfish)’ Parr *et al*. (2018)^34^	—
16. Off‐odors	Oxidized	oxidized Falanghina white wine	30 mL
	Cork taint	TCA	10 mL of a 11.7 ppb solution in distilled water
	Rotten eggs	hard‐boiled egg	10 g of chopped hard‐boiled egg
	Vinegar	wine vinegar	30 mL
	Smoked	smoked salt	1 g of smoked salt
	Cabbage	cabbage leaf	1 chopped leaf

During the first four training sessions, panelists were presented with 13–17 odor standards per session, encompassing two to five odor families. The standards were served in covered 80 mL disposable plastic cups, and panelists were tasked with recognizing the corresponding odor families or specific descriptors. Group discussions at the end of each session were conducted to avoid redundancy, clarify overlapping terms, and refine a consensual sensory vocabulary.

A fifth session focused on evaluating the intensity of odor descriptors using aqueous solutions of isoamyl acetate (Sigma‐Aldrich, St Louis, MO, USA) at five concentrations (100, 250, 400, 600, and 800 ppb) in a ranking test.

##### Training on taste/mouthfeel stimuli

Panelists were trained to identify taste and mouthfeel standards derived from established literature, with modifications.^29^, ^30^, ^35^ These standards represented key sensory dimensions critical for evaluating white wine: sweetness, acidity, bitterness, saltiness, drying, viscosity, and tingling. This latter descriptor has been defined in still wines as a ‘light, diffuse pins‐and‐needles’ sensation on the tongue.^36^

Over the first session, panelists were presented with six standards in aqueous solution (Table 2), each at a single high concentration to easily familiarize themselves with the corresponding sensory characteristics. Successively, the same standards were prepared at two concentration levels (Table 2), allowing panelists to practice recognition and discrimination at lower concentrations. The standards (40 mL) were served in covered 80 mL disposable plastic cups, and participants identified the associated descriptors and discussed their perceptions.

**Table 2:** Reference standards used to train the assessors in recognizing and distinguishing wine taste/mouthfeel properties

Product	Descriptor	Concentration (g L^−1^)		
		Easy level	Difficult level	
			1	2
Glucose	Sweetness	8	7	3
Caffeine	Bitterness	0.4	0.4	0.2
NaCl	Saltiness	5	2.5	1
Tannic acid	Astringency	2	0.8	0.4
Tartaric acid	Acidity	2	—	0.3
Malic acid	Acidity	‐	—	0.5

In the second session of taste/mouthfeel training, panelists conducted a five‐level intensity‐ranking test for sweetness (2–20 g L^−1^ glucose from J.T. Baker – Avantor, Radnor, PA, USA), bitterness (0.25–1.5 g L^−1^ caffeine from ACEF, Piacenza, Italy), viscosity (0–25 g L^−1^ carboxymethylcellulose from Enartis, Novara, Italy), tingling (10–100% sparkling water, San Pellegrino), saltiness (0.2–2.5 g L^−1^ NaCl from ACEF, Piacenza, Italy), acidity (0.2–1.5 g L^−1^ tartaric acid from Chem‐Lab, Eernegem, West‐Vlaanderen, Belgium), and drying (0.2–1.5 g L^−1^ tannic acid from J.T. Baker – Avantor).

Finally, four additional sessions familiarized panelists with the RATA method and the use of structured scales using real wine samples. Twenty representative wines from the study's sample set (one for each of the 18 monovarietal wines under investigation plus two commercial table wines) were presented (35 mL in black glasses coded with three‐digit identifiers). Panelists evaluated the wines on a numerical scale ranging from 1 (very low) to 5 (very high), with half‐point increments allowed. Water was provided for palate cleansing, and panelists waited at least 20 s between samples. As in a previous study,^30^ the performances of the trained judges were tested by a three‐way analysis of variance (ANOVA; Tukey, *P* ≤ 0.05) with three interactions: assessor × session, assessor × sample, and sample × session. Post‐session discussions aimed to harmonize the vocabulary for odor, taste, and mouthfeel descriptors and to refine the application of the intensity scale. Through these sessions, panelists achieved consensus on the sensory terminology and honed their ability to discriminate among white wines based on olfactory, taste, and mouthfeel attributes.

#### Sensory evaluation phase: RATA

Sensory analysis was conducted on the 246 monovarietal white wines applying the RATA method. Each wine was evaluated in duplicate, resulting in a total of 492 samples. Evaluations occurred across 41 sessions, with 12 wines assessed per session, using a fully randomized design for replicating analysis. Recovery sessions were organized within 2 days if a panelist was unavailable for a specific session. Samples (35 mL) were served in black wine glasses (INAO) coded with unique three‐digit identifiers and presented in randomized order among the judges. Sensory evaluations took place in individual booths maintained at room temperature (19 ± 2 °C). All participants evaluated the wines first by smelling and scoring odor intensities, using the consensually developed vocabulary, and then by tasting and scoring for taste/mouthfeel characteristics. Perceived intensities were scored on the same numerical scale employed during training.

### Data analysis

The sensory characterization of the wines was conducted through a statistical framework using XLSTAT (2020.5) software (Addinsoft, 2020) and its Sensory (2020.5) package. The analysis included the following steps:

- *Definition of the sensory lexicon*. A product characterization analysis considering the entire dataset of 246 wines was performed to establish a comprehensive sensory lexicon, including only the terms that showed significant differences among the wines at ANOVA. For each descriptor an ANOVA model was applied to check if the scores given by the judges were significantly different. The model (*P* ≤ 0.05) included an interaction factor: score (Y) = product (P) + assessor (A) + session (S) + product (P) × assessor (A). The judge effect was fixed as random.
- *Characterization and discrimination of varietal sensory attributes*. Descriptive and discriminative sensory attributes (taste, mouthfeel, and odor) specific to each variety were identified for each monovarietal wine through a product characterization analysis (ANOVA–Model: Y = P + A + S + P × A; *P* ≤ 0.05), allowing the identification of varietal sensory profiles and capturing the sensory diversity among wines.
- *Definition of the olfactory dimensions*. The first step was to establish relationships among the terms of the olfactory lexicon. The frequency with which each pair of terms was cited across wines was collated into a matrix, which served as a measure of proximity between descriptors. This matrix was then analyzed using hierarchical cluster analysis (HCA) (Ward's algorithm, Euclidean distance), highlighting homogeneous clusters of odors within the lexicon. Subsequently, a PCA was computed on the correlation matrices (Pearson, *P* < 0.05) of the mean intensities of olfactory descriptors of the monovarietal wines. These analyses aimed at defining the olfactory dimensions across the 18 wine types.
- *Generation of the sensory wheels as varietal sensory identity models*. Sensory wheels were developed to represent models of varietal sensory identity. These wheels were constructed using significant positive coefficients (*P* ≤ 0.05) from the ANOVA‐based product characterization analysis, incorporating both aroma and taste/mouthfeel descriptors. Each wheel offers a visual synthesis of a wine's sensory profile, highlighting the relative intensity of its most distinctive attributes.

## RESULTS AND DISCUSSION

As already highlighted, this study aimed to identify the sensory lexicon of 18 monovarietal Italian white wines with respect to the three main sensory modalities relevant to wine consumption (odor, taste, and mouthfeel). The final output is the identification of patterns of descriptive and discriminative attributes to build sensory wheels that can serve as reference models representing the sensory features of each monovarietal wine.

### Definition of the sensory lexicon

The first step aimed at identifying the sensory vocabulary capable of both describing and discriminating the 18 wine types. Through a product characterization analysis based on an ANOVA model (*P* ≤ 0.05), a sensory lexicon comprising 29 olfactory and seven taste/mouthfeel descriptors was established (Table 3). Descriptors with statistically significant *P*‐values were included and ranked in decreasing order of discriminant power for one or more wine types. Among these, the term ‘rose’ emerged as the most discriminative olfactory descriptor, while the descriptor ‘drying’ stood out among taste/mouthfeel properties. In contrast, ‘grapefruit’ and ‘tingling’ showed the lowest discriminative power for olfactory and taste/mouthfeel attributes, respectively. The defined olfactory lexicon encompasses a wide range of odor families, underscoring the aromatic complexity of the wines. Notably, the floral and exotic/tropical fruit families contain the highest number of highly discriminative descriptors, highlighting a rich aromatic diversity stemming from Italy's white wine biodiversity.

**Table 3:** Olfactory and taste/mouthfeel descriptors by decreasing discriminating power on the 18 monovarietal white wines. Associated test values and *P*‐values are also reported

Sensory modality	Descriptor	Test value	*P*‐value
Nose	Rose	14.65	<0.0001
	Mango	13.53	<0.0001
	Floral	12.94	<0.0001
	Off‐odors	12.08	<0.0001
	Fruity	10.38	<0.0001
	Mineral	8.95	<0.0001
	Passion fruit	8.71	<0.0001
	Thiolic (Sauvignon Blanc profile)	8.15	<0.0001
	Box tree/cat pee	7.42	<0.0001
	Vanilla	7.13	<0.0001
	Oxidized	7.00	<0.0001
	Woody	5.96	<0.0001
	Banana	5.50	<0.0001
	Sweet odors	5.33	<0.0001
	Citric	5.23	<0.0001
	Toasted	5.22	<0.0001
	Balsamic	4.88	<0.0001
	Tropical fruit	4.44	<0.0001
	Ethereal/alcohol	4.25	<0.0001
	Undergrowth	4.07	<0.0001
	Orange blossom	4.06	<0.0001
	Vegetal	3.94	<0.0001
	Smoked	3.48	<0.0001
	Lactic	3.46	<0.0001
	Spicy	3.00	0.0013
	Dried fruit	3.00	0.0014
	Aromatic herbs	2.80	0.0026
	Dehydrated fruit	2.66	0.0039
	Grapefruit	2.52	0.0058
Mouth	Drying	14.04	<0.0001
	Sweetness	12.66	<0.0001
	Acidity	8.02	<0.0001
	Viscosity	6.25	<0.0001
	Saltiness	3.28	0.0005
	Bitterness	3.18	0.0007
	Tingling	2.88	0.0020

The established sensory lexicon built on the whole set of 18 monovarietal wines served as the foundation for the subsequent sensory characterization and differentiation of each wine type. All further analyses were based on this vocabulary, ultimately aimed at defining the key traits and peculiarities of the sensory identity of the 18 monovarietal white wines.

### Taste and mouthfeel characterization of the wines

**Figure 1:**
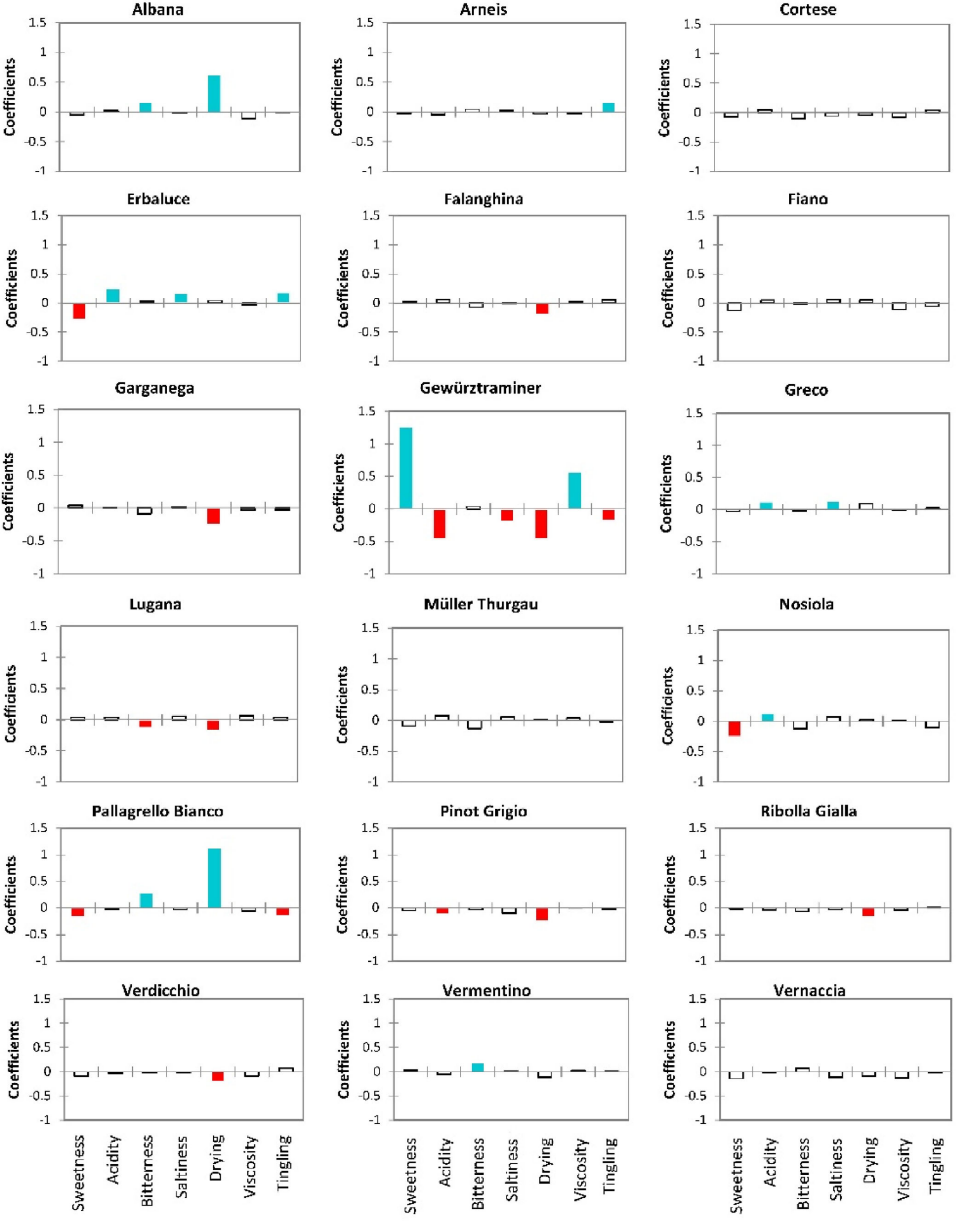
Graphs displaying the various coefficients of the chosen model for each combination product–taste/mouthfeel descriptor. The blue color is associated with coefficients that have a significant positive value, and the red color is associated with coefficients that have a significant negative value.

Based on the ANOVA outputs (Supporting Information, Table S2), Fig. 1 presents graphs displaying the model coefficients for the seven significant taste/mouthfeel descriptors (Table 3) for each monovarietal wine presented in alphabetic order: sweetness, acidity, bitterness, saltiness, drying, viscosity, and tingling. Model coefficients are numerical values that represent the influence or importance of each sensory attribute in characterizing each product, indicating how strongly that specific attribute is associated with a specific wine. Positive coefficients mean the attribute is more prominent or typical in that product, while negative coefficients suggest weaker associations with that product.^10^ In Fig. 1, for each monovarietal wine, blue bars represent positive model coefficients for descriptors whose adjusted means are significantly higher than the global mean, while red bars indicate negative model coefficients for descriptors with adjusted means significantly lower than the global mean.

According to the results (Fig. 1), the 18 monovarietal wines could be classified into four groups based on the number of taste and mouthfeel descriptors that, on average within each wine type, were perceived as more or less intense compared with the other wine types: (i) single‐attribute level, characterized by only one influencing descriptor: Arneis (positive model coefficient for tingling), Vermentino (positive for bitterness), and Falanghina, Garganega, Ribolla Gialla, and Verdicchio (negative model coefficient for drying); (ii) two‐attribute level, characterized by two influencing in‐mouth sensations: Albana (positive model coefficients for bitterness and drying), Greco (positive for acidity and saltiness), Lugana (negative model coefficients for bitterness and drying), Nosiola (positive model coefficient for acidity and negative for sweetness), and Pinot Grigio (negative for acidity and drying); (iii) four‐attribute level, characterized by four influencing sensory descriptors: Erbaluce (positive model coefficients for acidity, saltiness, and tingling, and a negative model coefficient for sweetness) and Pallagrello Bianco (positive model coefficients for bitterness and drying, and negative for sweetness and tingling); (iv) six‐attribute level, characterized by six influencing taste/mouthfeel sensations: Gewürztraminer, which is characterized by positive model coefficients for sweetness and viscosity, and negative for acidity, saltiness, drying, and tingling. Cortese, Fiano, Müller Thurgau, and Vernaccia monovarietal wines did not display any significant model coefficient for the taste/mouthfeel descriptors, suggesting that, according to the averages intensities measured, their in‐mouth features are less useful for the varietal recognizability.

### Olfactory characterization of the wines

#### Odor dimensions

**Figure 2:**
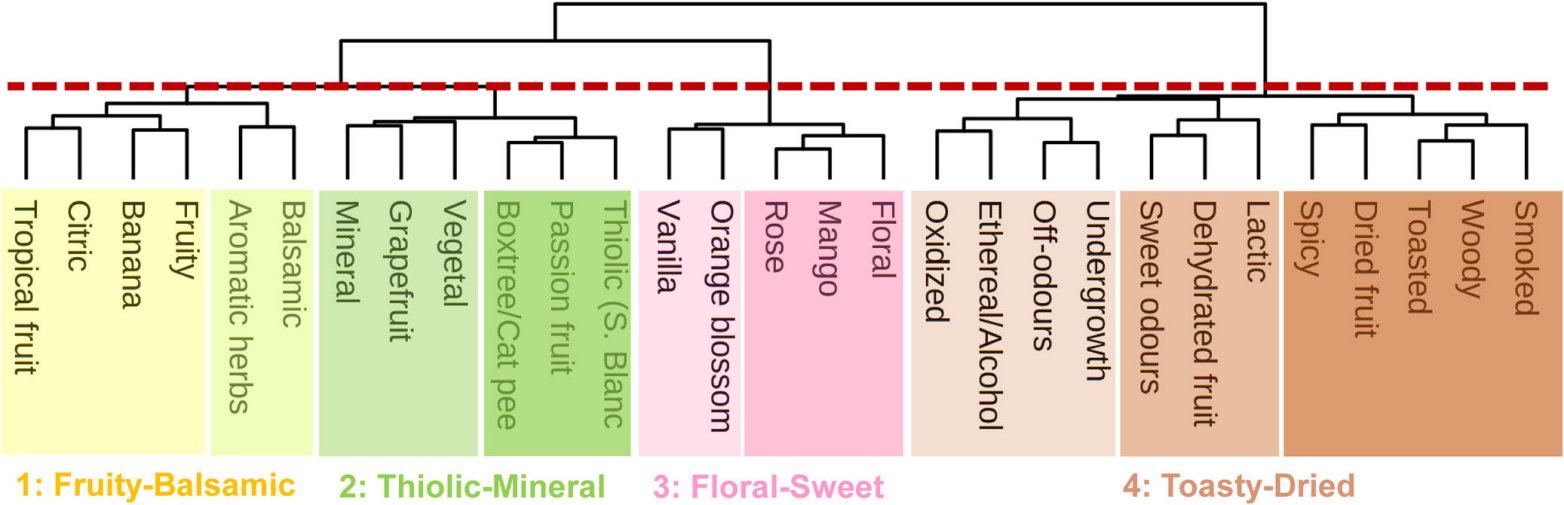
Hierarchical cluster analysis of odor descriptors based on co‐occurrence across the wine set, identifying four olfactory dimensions.

The co‐occurrence frequencies of olfactory descriptors across the wine samples were used to construct a proximity matrix, representing the associative strength between each pair of terms. This matrix was subsequently analyzed using HCA (Fig. 2), revealing distinct clusters of semantically related odors within the lexicon of 29 descriptors. This methodology revealed several logically consistent sub‐groupings of terms, which are discussed below in relation to the PCA results, which were helpful for choosing the truncation criterion of the dendrogram, and thus the cut‐off employed to define the number of clusters.

**Figure 3:**
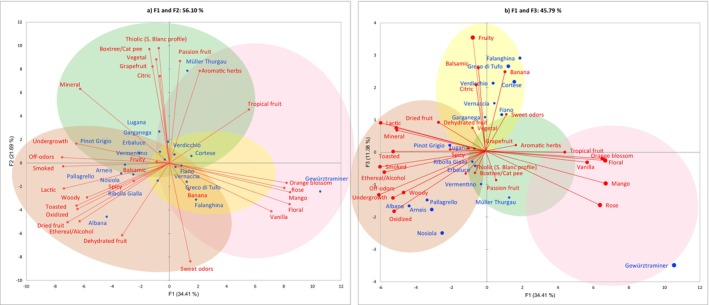
Principal component analysis biplots computed on the correlation matrices (Pearson, *P* < 0.05) of mean intensities for each wine (observations) of the 29 descriptors (variables) composing the olfactory lexicon. The colors of the circles correspond to the clusters identified by the hierarchical cluster analysis: yellow = fruity–balsamic cluster; green = thiolic–mineral cluster; pink = floral–sweet cluster; brick red = toasty–dried cluster.

The PCA computed on the correlation matrices (Pearson, *P* < 0.05) of mean intensities for each wine across the 29 descriptors composing the olfactory lexicon, shows the correlations of variables (odor descriptors) and observations (wine types), thereby revealing olfactory diversity and similarities within the sample set. Six components explained more than 80% of the total variance (84%), while the first six components satisfied Kaiser's criterion. After the examination of loadings, the first three components showing most of the variance (67.5%) are displayed in Fig. 3 as the most informative. All odor descriptors, except spices, show their highest squared cosines on the first three principal components, collectively representing 67.5% of the total variance. The biplot in Fig. 3(a) explains 56.1% of the variance across the first two components (34.4% and 21.7%, respectively). On F1, the descriptors mango, floral, orange blossom, rose, and vanilla were well correlated with each other and with Gewürztraminer, which displayed the highest squared cosine along the positive values of the component. In contrast, the highest squared cosines on F1 negative semiaxis are shown by Albana, Arneis, Pallagrello Bianco, and Nosiola, together with the descriptors smoked, lactic, ethereal/alcoholic, undergrowth, toasted, dried fruits, woody, and off‐odors, including oxidized. On F2 the descriptors aromatic herbs, passion fruit, thiolic, box tree/cat pee, vegetal, grapefruit and citrus are well correlated with each other and with Müller Thurgau. This latter wine shows the highest squared cosine along the positive values of the component, followed by Lugana and Garganega. In opposition, the variable dehydrated fruit has the best projection on the F2 negative semiaxis together with sweet odors, here showing the highest squared cosine. Tropical fruit and mineral almost behave as bisectors of the first and forth quadrants, respectively. Together with sweet odors, they are well projected on the components and, at the same time, are less correlated with the three main groups of variables highlighted in the biplot. The biplot in Fig. 3(b) presents the F1 and F3 components. F3 contributes 11.4% of the variance and, collectively, F1 and F3 explain 45.8% of the total variance. On this component the fruity, balsamic, and banana variables show the highest values of squared cosines, well correlated with Falanghina and Greco di Tufo wines together with Cortese, Verdicchio, and Fiano.

Globally, along the first three principal components, a tridimensional area is defined across four directions. With their highest values of quadratic cosines, Müller Thurgau, Gewürztraminer, Albana, and Falanghina can be considered the most representative wines along these four olfactory directions and delineate the vertices of a space within and around which the other wines are distributed.

These results suggested a criterion for truncating the HCA dendrogram (Fig. 2), showing that the odor descriptors grouped into four main clusters, which correspond closely to the four directions resulting from the PCA. The truncation criterion led to the association of descriptors that seem both semantically coherent and well represented within the sample set of Italian wines. The first cluster comprises two subgroups: (i) fruity, banana, citrus, and tropical fruit; and (ii) balsamic and aromatic herbs. The second cluster also includes two subgroups: (i) vegetal, grapefruit, and mineral; and (ii) thiolic notes (Sauvignon Blanc‐like profile), passion fruit, and box tree/cat pee. The third cluster consists of: (i) orange blossom and vanilla, and (ii) floral, rose, and mango. Finally, the fourth cluster encompasses three subgroups: (i) undergrowth, off‐flavors, oxidized, and ethereal/alcoholic, (ii) lactic, dehydrated fruit, and sweet odors, and (iii) smoky, woody, toasted, dried fruit, and spices.

Altogether, the results from HCA and PCA identified four ‘odor dimensions’ to which the 18 monovarietal Italian white wines belong. These dimensions describe the sensory features and diversity of the wines. Based on the descriptors composing the four clusters, the odor dimensions were arbitrarily labeled as: fruity–balsamic, thiolic–mineral, floral–sweet and toasty–dried, with the latter including odor faults.

Several of the co‐occurrences observed across the four dimensions appear to be consistent with some previous findings reported in the literature. The clustering aligns well with some sensory vectors proposed by Ferreira *et al*.^37^ as sensory outputs linked to pools of specific volatiles. For example, the third cluster groups together descriptors such as floral, rose‐like, sweet, and orange blossom that fall into the ‘floral’ vector. The first and second clusters, in turn, may be interpreted as interconnected segments along a broader sensory continuum ranging from ‘fresh‐fruity’ to ‘green’, including descriptors such as fruity, banana, tropical fruit (e.g., passion fruit), citrus (e.g., grapefruit), box tree, vegetal, and fresh classified within the fruity, citrus‐green, and vegetal vectors, representing some important aroma categories in young white wines. Heymann *et al*.^38^ observed that wine professionals and trained panelists often associated minerality with citrus, fresh, and grassy aromas. Similarly, the thiol character has been linked to descriptors such as passion fruit, box tree, cat pee, grapefruit, and vegetal notes^39^ in Sauvignon Blanc wines. Lastly, the fourth cluster, labeled ‘toasty–dried’, comprises descriptors such as smoky, woody, toasted, dried fruit, spices, and some off‐notes (e.g., oxidized, ethereal/alcoholic), which are consistent with the ‘woody/spices’ vector.

Looking ahead, it will be interesting to assess in a subsequent analysis whether the correlations among descriptors observed in the clustering are supported by chemical data on volatile composition, and therefore interpreting the observed clustering patterns and their correspondence with molecular drivers.

#### Olfactory description and discrimination

**Figure 4:**
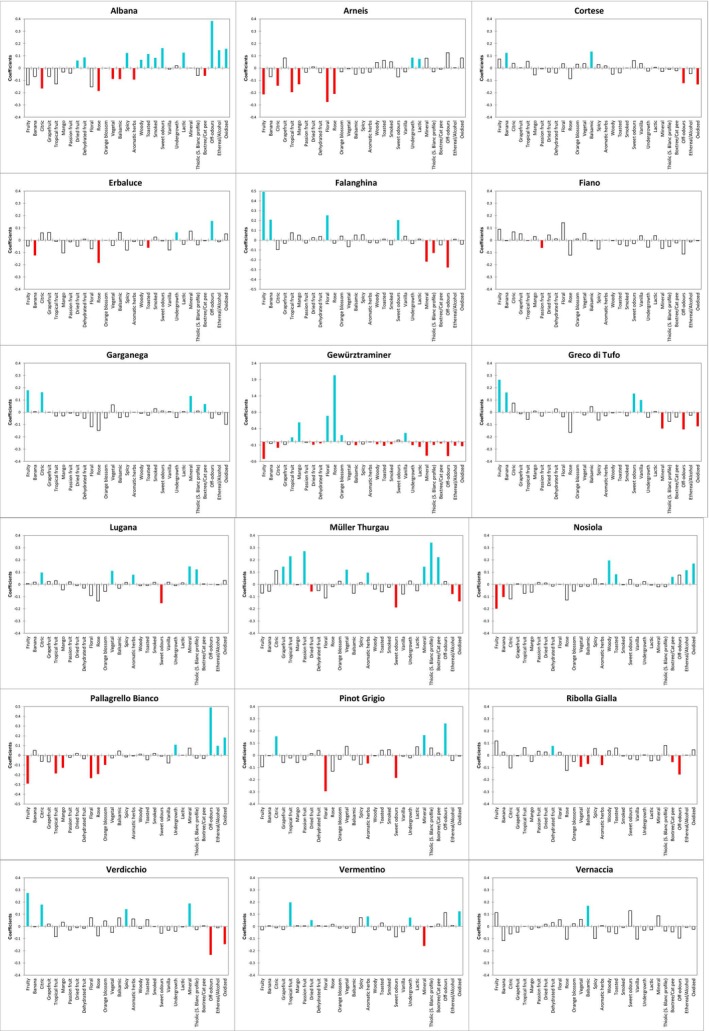
Graphs displaying the various coefficients of the chosen model for each combination product–olfactory descriptor. The blue color is associated with coefficients that have a significant positive value, and the red color is associated with coefficients that have a significant negative value.

As previously done for taste and mouthfeel properties, a product characterization analysis was conducted to identify attributes that were both olfactory descriptive (Fig. 4) and discriminating (Supporting Information, Table S3). Figure 4 provides a comprehensive description of the olfactory profiles of each monovarietal wine and displays positive and negative model coefficients for each combination product–descriptor.

Supporting Information, Table S3, shows the adjusted means for each product–descriptor combination. Colors indicate a significant positive effect (blue) or a significant negative effect (red). The *P*‐values (*P* ≤ 0.05) are also displayed, with descriptors ordered from the most to the least discriminating.

The patterns highlight a broad olfactory diversity, as well as some similarities, across the 18 monovarietal white wines. The graphs describe each wine based on both the odors that characterize it and those that are absent from that specific wine type.

Overall, the results reveal broad olfactory diversity. Gewürztraminer displays the highest number of positive and negative discriminating odor descriptors. Notably, rose, floral, mango, tropical fruit, vanilla and orange blossom are its six positively discriminating attributes, with the first four showing the highest mean intensities. Across all wines, Gewürztraminer stands out for its floral and tropical character. Falanghina wines are similarly characterized by prominent floral odors, alongside fruity, sweet, and banana notes. Greco di Tufo shares many of these attributes but can be differentiated from Falanghina for a non‐significant floral character and the presence of a vanilla note. The banana descriptor is also relevant for Cortese wines, which share a balsamic character with Vernaccia di San Gimignano and Vermentino. Both Cortese and Vernaccia, along with Fiano, exhibit a general fruity character (even if not statistically significant). Fruity odors, combined with mineral and citrus notes, characterize Garganega and Verdicchio wines. Garganega also exhibits a box tree/cat pee aroma, whereas Verdicchio shows a spiced scent. Mineral notes, often associated with citrus odors, are also important for Pinot Grigio (also sulfurous) and Lugana. Lugana shares aromatic herbs, thiolic, vegetal, and mineral descriptors with Müller Thurgau, which is further strongly linked to tropical fruit, passion fruit, grapefruit and box tree/cat pee aromas, as previously observed by Carlin *et al*.^25^ Globally, all these wines are primarily related to descriptors belonging to floral–sweet, fruity–balsamic, and thiolic–mineral odor dimensions, as highlighted above by HCA clusters and PCA (Figs 2 and 3). Conversely, descriptors clustered within the olfactory dimension labeled ‘toasty–dried’ are more relevant for the remaining monovarietal wines. Besides some odor taints, Albana, Pallagrello Bianco, and Erbaluce wines are consistently characterized by undergrowth notes. Ethereal/alcoholic notes are particularly relevant for Albana and Pallagrello Bianco, while sweet odors dominate Albana wines, which also have spices, lactic, toasted, dried fruit, woody, and dehydrated fruit aromas. Arneis shares undergrowth and lactic odors with Albana, and dehydrated fruit note with Ribolla Gialla, while oxidized, woody, ethereal/alcoholic, and toasted aromas associated with Nosiola, which also has a box tree/cat pee note.

It is well established that aromas can influence one another through masking or enhancement effects.^40^, ^41^, ^42^ Therefore, the presence – as well as the absence – of specific odor notes may result not only from the chemical composition of the wine, but also from perceptual interactions occurring between/among sensory‐active compounds. The sensory patterns observed in our dataset may reflect such interactions between descriptors. Wines dominated by the toasty–dried dimension (i.e., Albana, Pallagrello Bianco, Erbaluce, Nosiola, and Arneis) display strong smoky, woody, toasted, lactic, and dehydrated fruit notes, as well as some odor faults. These same wines are lacking in descriptors associated with the fruity, green, or fresh odor domains. Based on previous results^43^, ^44^ it could be hypothesized that the presence of toasty–dried attributes may have contributed to the perceptual masking of fresher or fruitier notes. The hypothesis has to be verified by studying the aromatic chemical composition. Perceptual interactions between mineral and floral–fruity notes have been previously reported,^45^, ^46^ suggesting that mineral aromas, for example those driven by compounds such as methanethiol, can exert a suppressive effect on fruity and floral perception in white wines. In line with this, our findings suggest that wines characterized by floral and fruity notes (i.e., Gewürztraminer, Vermentino, Falanghina, Greco di Tufo, and Verdicchio) tend to lack mineral, thiolic, or faulty odor notes (e.g., oxidized), reinforcing the idea of mutual suppression between certain odor dimensions. Lastly, the more thiolic, mineral, and vegetal wines (i.e., Pinot Grigio, Lugana, and Müller Thurgau) lack descriptors associated with sweet or floral odors.

These observations suggest that the sensory characterization of a specific wine should not be limited to the descriptors that are present but should also consider those that are absent. In fact, the absence of specific odor notes may carry important information, particularly in the context of perceptual interactions, and can provide valuable insights into the mechanisms by which certain aromas are suppressed or modulated within complex odor mixtures. Further investigation is needed also considering the chemical data on odorants and ultimately helping in unveiling the link between wine composition and sensory features.

### Generation of sensory wheels

**Figure 5:**
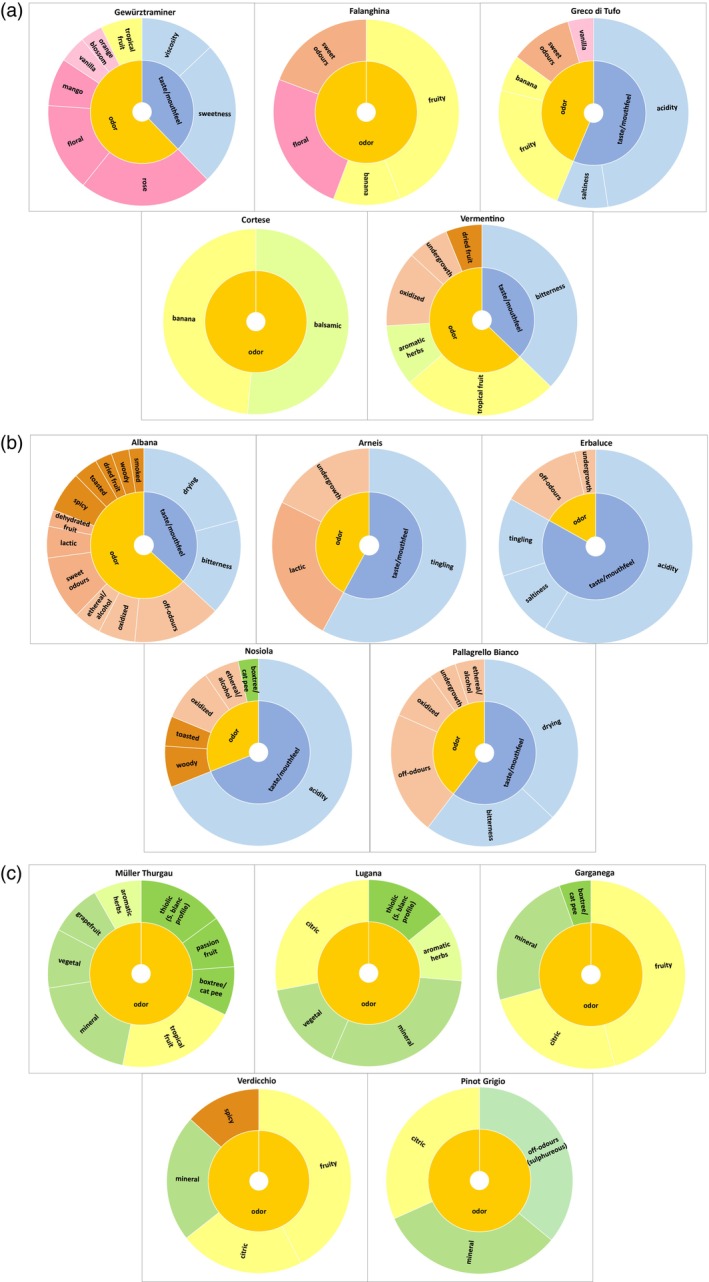
Monovarietal sensory wheels. They were created based on the values of the significant positive coefficients of both flavor (taste/mouthfeel) and smell (odor) descriptors. The larger the area underpinning a descriptor, the higher is its coefficient and therefore its sensory relevance. Odor descriptors are associated with the same colors used in Figs 2 and 3, while for in‐mouth sensations blue shades are used.

The information gathered above was used to generate sensory wheels, serving as ‘sensory identity models’ (Fig. 5(a)–(c)). These were based on the significant positive coefficients of both taste/mouthfeel and odor descriptors reported in Fig. 1 and Supporting Information, Table S2, as well as in Fig. 4 and Supporting Information, Table S3. The larger the area underpinning a descriptor, the higher its coefficient and, consequently, its sensory relevance. Odor descriptors were color‐coded consistently with Fig. 2 (HCA) and 3 (PCA), while for in‐mouth sensations blue shades were used. The sensory wheels thus provide a comprehensive and easy‐to‐read overview of the descriptors useful to describe the main characteristics that one can expect to experience when tasting each type of wine, also giving an idea of the olfactory dimension/s to which it belongs. Based on the wheels, wines are distinguishable by two groups: those whose most relevant sensory traits are only related to smell features (Müller Thurgau, Lugana, Garganega, Pinot Grigio, Verdicchio, Cortese, Falanghina) and those showing a combination of both smell and of taste and/or mouthfeel sensations as relevant descriptors (Albana, Arneis, Erbaluce, Pallagrello Bianco, Nosiola, Vermentino, Gewürztraminer, Greco di Tufo) helpful for their sensory recognition.

With a prevalence of floral notes and a key dominating rose‐like smell that confirms previous findings, the sensory wheel of Gewürztraminer shows that it is the most representative of the floral–sweet dimension (Fig. 5(a)). This observation is consistent with the well‐documented sensory and compositional features of Gewürztraminer. In fact, Traminers typically exhibit elevated concentrations of terpenoids such as geraniol and nerol, along with the characteristic presence of *cis*‐ and *trans*‐rose oxide, that contribute to its intense rose‐like aromatic character.^47^, ^48^ The Gewürztraminer wheel also reveals a more diverse sensory interplay, combining olfactory, taste, and mouthfeel characteristics. This wine is distinguished by its notable sweetness and viscosity – attributes likely stemming from its higher average sugar content compared to other wines (Supporting Information, Table S1). A more general floral character is also relevant for Falanghina (Fig. 5(a)). Although this wine demonstrates a globally less complex sensory profile, marked by fewer discriminating descriptors and a lack of significant in‐mouth features, it is highly representative of the fruity dimension. A key banana note defines its aromatic character, a trait shared with Greco di Tufo (Fig. 5(a)). While both wines align with the fruity dimension, Falanghina maintains its floral distinction, which is not as pronounced in Greco di Tufo. Complementing its fruity–sweetness, sourness and saltiness also contribute to Falanghina's taste balance. The wheel of Cortese (Fig. 5(a)) links to the fruity dimension with a simple pattern showing a balance between banana and balsamic herb notes. Also, in the Vermentino aroma wheel (Fig. 5(a)), a balsamic character is paired with a fruity one – this time marked by tropical notes, accompanied by a lack of freshness reflected in dried fruit, undergrowth, and oxidized aromas, along with a dominant bitter taste. A similar lack of freshness, likely linked to the absence of fruitiness, is common in all the wheels of wines dominated by descriptors belonging to the dimension labelled ‘toasty–dried’ (Fig. 5(b)), except for Nosiola, showing a small but significant contribution of a box tree/cat pee smell. The wheel of Albana shows the most complex pattern within the ‘toasty–dried’ dimension (Fig. 5(b)). An interesting observation is that all wines characterized by descriptors within this olfactory dimension also incorporate in‐mouth features into their sensory profiles and stand in opposition to wines primarily defined by a mineral character, whose sensory wheels do not include in‐mouth characteristics. Minerality is often associated with thiolic notes (meaning a Sauvignon Blanc‐like profile, sometimes including a specific cat pee odor) and associated aromas such as vegetal (sometimes including aromatic herbs), citrus (sometimes including grapefruit) and tropical (sometimes including passion fruit).^34^, ^38^, ^45^, ^46^ The most representative wine of this ‘thiolic–mineral’ dimension (Fig. 5(c)) is Müller Thurgau, showing a complex odor wheel, followed by Lugana and Garganega, which, together with Verdicchio, has a dominating fruity character, with citrus notes that together with minerality, includes also Pinot Grigio in this olfactory dimension (Fig. 5(c)). This thiolic aspect was confirmed by the volatile fraction attributable to thiols.^25^ These authors previously observed that volatile thiols were detected across all cultivars, although higher concentrations of 3‐sulfanylhexan‐1‐ol (3‐SH) were found in Lugana, Müller‐Thurgau, and Verdicchio. Notably, Müller‐Thurgau wines exhibited the highest levels of 4‐methyl‐4‐sulfanylpentan‐2‐one (4‐MSP), which was strongly correlated with the odor descriptors passion fruit and box tree/cat urine, thus highlighting its key olfactory role in the varietal aroma.

Overall, the 15 sensory wheels presented in Fig. 5 illustrate the diversity and uniqueness of the sensory profiles associated with each wine, highlighting the richness of Italy's native white grape varieties. These models may serve as useful reference tools for winemakers, sellers, and consumers, providing a clear and structured overview of the key sensory features of each monovarietal wine. Since the wines analyzed were commercial products – rather than experimental samples produced under a standardized protocol – the significant descriptors identified are likely to reflect the typical characteristics of the grape variety and its denomination. Indeed, they pop out as common features of the specific monovarietal wine despite differences in winemaking. Beyond their practical utility for communication and marketing, these wheels may also support dissemination efforts, prove valuable for researchers, and serve as a useful tool for sensory quality control activities carried out within PDO appellation systems (e.g., Valoritalia). They offer a starting point for investigating correlations and interactions between chemical and sensory data and may facilitate the study of perceptual and cross‐modal interactions that contribute to the overall sensory experience of wine tasting.

For Fiano, Vernaccia, and Ribolla Gialla, as the different steps of the sensory characterization study highlighted only one descriptor with positive or negative model coefficient for each wine type (passion fruit as negative, balsamic and dehydrated fruit as positive, respectively), the results were not sufficiently stable or informative to build reliable models useful to generate the corresponding sensory wheels (Supporting Information, Table S3). This limitation could be addressed by applying the same experimental design to a larger sample set of these three monovarietal wines.

The model sensory wheels developed for those wines belonging to the olfactory dimension labeled ‘toasty–dried’ should be further verified before validation, due to the potential influence of olfactory taints that could impact the results.

On the other hand, for most of the wines it was possible to generate sensory wheels as stable models that could be validated by researchers through next sensory and compositional studies on the same wine types and even in comparison with profiles developed by expert panels composed of experienced enologists.

## CONCLUSIONS

This study presents a detailed sensory mapping of 18 Italian monovarietal white wines, integrating olfactory, taste, and mouthfeel features into structured identity models. A statistically grounded sensory lexicon was developed using ANOVA, followed by multivariate analyses (HCA, PCA), which identified four main olfactory dimensions: fruity–balsamic, thiolic–mineral, floral–sweet, and toasty–dried. These, combined with discriminant taste and mouthfeel descriptors, formed the basis for sensory wheels that encapsulate each wine's unique sensory signature and support their differentiation within the broader context of Italian white wines.

Findings indicate that sensory recognizability in some wines depends not only on olfactory traits but also on their integration with gustatory and mouthfeel sensations. The resulting models offer a reference framework for profiling Italian white wines from native varieties, aiding their communication and valorization. To date, no sensory data exist in the literature for Nosiola, Pallagrello Bianco, Erbaluce, and Albana.

The developed wheels are valuable tools for producers, consumers, and researchers. They help convey sensory expectations, support recognition of lesser‐known varieties, and contribute to defining quality benchmarks. Scientifically, they offer a foundation for exploring chemical relationships and perceptual interactions. The dataset enables investigation of olfactory and in‐mouth interactions, enhancing understanding of sensory diversity in Italian monovarietal wines. Within the D‐WINES project, ongoing research is integrating these sensory data with chemical data to identify molecular markers linked to sensory identity.

These insights support producers in refining quality management and production guidelines, improving communication and promotion of Italian white wines to national and international consumers.

## CONFLICT OF INTEREST

The authors declare that they have no known competing financial interests or personal relationships that could have appeared to influence the work reported in this paper.

## FUNDING INFORMATION

The project ‘The aroma diversity of Italian white wines. Study of chemical and biochemical pathways underlying sensory characteristics and perception mechanisms for developing models of precision and sustainable enology’ was funded by Ministry of Education, University and Research under the PRIN 2017 grant (Prot. 2017RXFFRR).

## Supporting information


**Table S1.** Mean, minimum, and maximum value of basic enological parameters in the analyzed wines.
**Table S2.** Descriptive and discriminative taste and mouthfeel characteristics specific to each monovarietal wine. Descriptor, estimated mean, and *P*‐value for each combination product–taste/mouthfeel descriptor are reported.
**Table S3.** Descriptive and discriminative olfactory characteristics specific to each monovarietal wine. Descriptor, estimated mean, *P*‐value for each combination product–olfactory descriptor are reported. The column ‘Cluster’ refers to the HCA in Fig. 2.

## Data Availability

The data that supports the findings of this study are available in the supplementary material of this article.
