# Supplementary material for: Definition of a sensory lexicon and development of sensory wheels of eighteen monovarietal Italian white wines
Source: J Sci Food Agric. 2026 Jan 28;106(6):3745–59. doi: 10.1002/jsfa.70465 (PMC12988708; doi:10.1002/jsfa.70465)
Supplement: Supplementary file 1 — Table S1. Mean, minimum, and maximum value of basic enological parameters in the analyzed wines. Table S2. Descriptive and discriminative taste and mouthfeel characteristics specific to each monovarietal wine. Descriptor, estimated mean, and P‐value for each combination product–taste/mouthfeel descriptor are reported. Table S3. Descriptive and discriminative olfactory characteristics specific to each monovarietal wine. Descriptor, estimated mean, P‐value for each combination product–olfactory descriptor are reported. The column ‘Cluster’ refers to the HCA in Fig. 2. [file JSFA-106-3745-s001.docx]

| **Table S1.** Mean, minimum and maximum value of basic enological parameters in the analyzed wines. | | | | | | | | |  |  |  |  |  |
| --- | --- | --- | --- | --- | --- | --- | --- | --- | --- | --- | --- | --- | --- |
|  |  | **Lactic acid (g/L)** | **Malic acid (g/L)** | **Tartaric acid (g/L)** | **Total acidity (g/L tartaric acid)** | **pH** | **Volatile acidity (g/L acetic acid)** | **Alcohol content (% vol)** | **Residual reducing sugars (g/L)** | **Total dry extract (g/L)** | **Non-reducing extract (g/L)** | **Free sulfur dioxide (mg/L)** | **Total sulfur dioxide (mg/L)** |
| **Albana** | Mean | 0.36 | 1.41 | 2.36 | 5.92 | 3.34 | 0.41 | 13.87 | 2.48 | 24.3 | 22.2 | 11 | 66 |
|  | Min | 0.10 | 0.10 | 1.30 | 4.11 | 3.19 | 0.27 | 12.76 | 1.00 | 20.8 | 19.6 | 5 | 28 |
|  | Max | 1.20 | 2.30 | 3.20 | 7.37 | 3.61 | 0.62 | 14.82 | 7.40 | 35.6 | 28.2 | 19 | 97 |
| **Arneis** | Mean | 0.46 | 1.33 | 2.10 | 5.26 | 3.33 | 0.33 | 13.43 | 1.91 | 20.1 | 18.5 | 18 | 74 |
|  | Min | 0.10 | 0.10 | 1.70 | 4.32 | 3.24 | 0.26 | 12.73 | 1.00 | 18.0 | 16.9 | 5 | 37 |
|  | Max | 1.60 | 2.10 | 2.40 | 5.88 | 3.47 | 0.47 | 14.49 | 3.60 | 22.8 | 20.3 | 44 | 121 |
| **Cortese** | Mean | 0.26 | 1.54 | 1.98 | 5.37 | 3.25 | 0.31 | 12.66 | 2.53 | 20.8 | 18.4 | 22 | 77 |
|  | Min | 0.10 | 0.50 | 1.70 | 4.89 | 3.17 | 0.22 | 12.02 | 1.00 | 18.0 | 16.9 | 9 | 51 |
|  | Max | 1.00 | 2.20 | 2.40 | 6.29 | 3.38 | 0.46 | 13.39 | 5.10 | 24.8 | 21.4 | 40 | 130 |
| **Erbaluce** | Mean | 0.43 | 2.01 | 2.47 | 6.46 | 3.22 | 0.35 | 12.49 | 1.73 | 22.5 | 21.0 | 19 | 76 |
|  | Min | 0.10 | 0.40 | 1.80 | 5.43 | 3.05 | 0.11 | 11.80 | 1.00 | 20.0 | 19.3 | 5 | 21 |
|  | Max | 1.50 | 3.80 | 3.30 | 8.46 | 3.35 | 0.50 | 13.22 | 4.00 | 26.0 | 25.0 | 48 | 123 |
| **Falanghina** | Mean | 0.21 | 2.75 | 1.95 | 6.24 | 3.42 | 0.36 | 13.46 | 1.81 | 22.9 | 21.6 | 13 | 75 |
|  | Min | 0.10 | 0.20 | 1.20 | 4.75 | 3.23 | 0.28 | 12.98 | 1.00 | 21.0 | 19.3 | 5 | 42 |
|  | Max | 1.50 | 4.00 | 2.90 | 6.95 | 3.85 | 0.41 | 14.50 | 5.30 | 25.9 | 25.4 | 31 | 103 |
| **Fiano** | Mean | 0.28 | 1.75 | 2.25 | 5.98 | 3.33 | 0.39 | 13.53 | 1.58 | 21.4 | 20.3 | 14 | 66 |
|  | Min | 0.10 | 0.10 | 1.60 | 5.55 | 3.21 | 0.27 | 12.84 | 1.00 | 19.6 | 18.3 | 5 | 39 |
|  | Max | 1.50 | 2.50 | 2.90 | 6.38 | 3.47 | 0.75 | 14.34 | 2.80 | 23.8 | 22.1 | 25 | 104 |
| **Garganega** | Mean | 0.27 | 1.49 | 2.53 | 5.50 | 3.32 | 0.29 | 12.47 | 3.71 | 24.1 | 20.4 | 19 | 78 |
|  | Min | 0.10 | 0.30 | 2.00 | 4.93 | 3.16 | 0.16 | 11.87 | 1.00 | 20.4 | 17.8 | 5 | 36 |
|  | Max | 0.70 | 2.30 | 3.20 | 6.02 | 3.45 | 0.44 | 13.05 | 10.10 | 31.2 | 23.4 | 39 | 108 |
| **Gewürztraminer** | Mean | 0.18 | 1.64 | 1.62 | 4.92 | 3.57 | 0.42 | 14.71 | 5.79 | 28.4 | 22.6 | 22 | 87 |
|  | Min | 0.10 | 0.50 | 1.20 | 4.11 | 3.29 | 0.33 | 13.10 | 1.00 | 19.4 | 19.2 | 7 | 54 |
|  | Max | 1.00 | 2.10 | 2.20 | 6.53 | 3.76 | 0.56 | 15.67 | 17.70 | 45.0 | 27.3 | 32 | 136 |
| **Greco di Tufo** | Mean | 0.35 | 1.94 | 2.25 | 6.24 | 3.31 | 0.38 | 13.38 | 1.85 | 22.9 | 21.2 | 14 | 71 |
|  | Min | 0.10 | 0.10 | 1.60 | 5.57 | 3.11 | 0.25 | 12.54 | 1.00 | 19.8 | 19.8 | 5 | 44 |
|  | Max | 1.80 | 2.90 | 2.90 | 7.32 | 3.42 | 0.58 | 14.30 | 4.80 | 25.6 | 22.9 | 23 | 115 |
| **Lugana** | Mean | 0.22 | 1.65 | 2.47 | 5.93 | 3.30 | 0.32 | 12.87 | 4.26 | 26.1 | 21.9 | 12 | 72 |
|  | Min | 0.10 | 0.80 | 1.70 | 4.96 | 3.13 | 0.21 | 12.23 | 1.00 | 21.7 | 20.0 | 5 | 42 |
|  | Max | 1.00 | 2.30 | 3.30 | 6.68 | 3.45 | 0.45 | 13.76 | 7.50 | 30.3 | 24.1 | 23 | 108 |
|  |  |  |  |  |  |  |  |  |  |  |  |  |  |
|  |  |  |  |  |  |  |  |  |  |  |  |  |  |
| **Table S1. Continued** | |  |  |  |  |  |  |  |  |  |  |  |  |
| **Müller Thurgau** | Mean | 0.11 | 2.07 | 2.17 | 5.80 | 3.27 | 0.31 | 12.39 | 2.23 | 21.6 | 19.5 | 18 | 74 |
|  | Min | 0.10 | 0.90 | 1.40 | 5.22 | 3.19 | 0.24 | 11.88 | 1.00 | 18.8 | 17.5 | 11 | 53 |
|  | Max | 0.20 | 2.50 | 2.70 | 6.48 | 3.38 | 0.43 | 13.32 | 4.10 | 24.6 | 21.4 | 27 | 95 |
| **Nosiola** | Mean | 0.58 | 1.57 | 2.09 | 6.03 | 3.30 | 0.33 | 12.18 | 1.96 | 22.8 | 21.1 | 18 | 71 |
|  | Min | 0.10 | 0.10 | 1.70 | 4.64 | 3.14 | 0.28 | 10.86 | 1.00 | 19.3 | 17.5 | 5 | 47 |
|  | Max | 1.80 | 2.80 | 2.40 | 7.36 | 3.56 | 0.46 | 13.33 | 4.10 | 25.9 | 23.3 | 34 | 92 |
| **Pallagrello Bianco** | Mean | 0.35 | 1.43 | 2.57 | 5.66 | 3.33 | 0.35 | 13.42 | 1.72 | 20.8 | 19.8 | 12 | 71 |
|  | Min | 0.10 | 0.40 | 2.10 | 4.80 | 3.13 | 0.25 | 12.58 | 1.00 | 17.8 | 17.8 | 5 | 37 |
|  | Max | 1.60 | 2.40 | 3.40 | 6.37 | 3.52 | 0.46 | 14.27 | 4.40 | 24.6 | 23.6 | 37 | 119 |
| **Pinot Grigio** | Mean | 0.28 | 1.63 | 2.18 | 5.44 | 3.34 | 0.25 | 12.55 | 3.48 | 24.3 | 20.8 | 21 | 82 |
|  | Min | 0.10 | 0.80 | 1.60 | 5.02 | 3.14 | 0.22 | 11.91 | 1.20 | 19.0 | 17.8 | 7 | 55 |
|  | Max | 0.80 | 2.60 | 2.70 | 6.26 | 3.48 | 0.30 | 13.13 | 6.70 | 28.7 | 23.5 | 36 | 121 |
| **Ribolla Gialla** | Mean | 0.32 | 1.84 | 2.03 | 5.62 | 3.36 | 0.32 | 12.75 | 2.33 | 22.9 | 20.8 | 21 | 79 |
|  | Min | 0.10 | 0.30 | 1.50 | 4.80 | 3.17 | 0.24 | 12.19 | 1.00 | 20.1 | 19.9 | 6 | 45 |
|  | Max | 1.20 | 3.60 | 2.80 | 6.67 | 3.46 | 0.56 | 13.50 | 7.40 | 28.4 | 21.8 | 34 | 119 |
| **Verdicchio** | Mean | 0.41 | 1.40 | 2.26 | 5.68 | 3.33 | 0.40 | 13.27 | 2.15 | 23.0 | 21.1 | 21 | 83 |
|  | Min | 0.10 | 0.10 | 1.90 | 5.01 | 3.16 | 0.29 | 12.47 | 1.00 | 20.4 | 19.0 | 5 | 56 |
|  | Max | 1.50 | 2.00 | 3.00 | 6.29 | 3.41 | 0.46 | 13.80 | 5.90 | 28.7 | 22.8 | 35 | 111 |
| **Vermentino** | Mean | 0.21 | 1.69 | 2.12 | 5.38 | 3.41 | 0.28 | 13.76 | 1.28 | 23.0 | 22.5 | 13 | 84 |
|  | Min | 0.10 | 1.10 | 1.50 | 4.73 | 3.23 | 0.12 | 12.27 | 1.00 | 20.1 | 18.6 | 5 | 59 |
|  | Max | 0.50 | 2.60 | 2.80 | 6.48 | 3.84 | 0.40 | 15.41 | 2.90 | 27.8 | 27.8 | 35 | 125 |
| **Vernaccia** | Mean | 0.18 | 1.59 | 2.45 | 5.60 | 3.32 | 0.31 | 12.85 | 1.24 | 20.8 | 20.3 | 17 | 70 |
|  | Min | 0.10 | 1.30 | 1.70 | 4.76 | 3.20 | 0.24 | 12.36 | 1.00 | 19.2 | 19.0 | 8 | 49 |
|  | Max | 0.30 | 2.00 | 2.80 | 6.43 | 3.44 | 0.42 | 13.49 | 2.20 | 22.3 | 21.8 | 31 | 90 |

| **Table S2.** Descriptive and discriminative taste and mouthfeel characteristics specific to each monovarietal wine. Descriptor, Estimated mean, p-value for each combination product-taste/mouthfeel descriptor are reported. | | | | | | | | | | | | | | | | | |  |
| --- | --- | --- | --- | --- | --- | --- | --- | --- | --- | --- | --- | --- | --- | --- | --- | --- | --- | --- |
| **Albana** | | | **Arneis** | | | **Cortese** | | | **Erbaluce** | | | **Falanghina** | | | **Fiano** | | |  |
| **Descriptor** | **Estimated mean** | **p-value** | **Descriptor** | **Estimated mean** | **p-value** | **Descriptor** | **Estimated mean** | **p-value** | **Descriptor** | **Estimated mean** | **p-value** | **Descriptor** | **Estimated mean** | **p-value** | **Descriptor** | **Estimated mean** | **p-value** |  |
| Drying | 1.374 | < 0.0001 | Tingling | 0.704 | 0.006 | Bitterness | 0.786 | 0.121 | Acidity | 3.246 | < 0.0001 | Drying | 0.546 | 0.002 | Sweetness | 1.063 | 0.073 |  |
| Bitterness | 1.057 | 0.017 | Acidity | 2.957 | 0.404 | Viscosity | 0.628 | 0.174 | Saltiness | 0.624 | 0.001 | Acidity | 3.061 | 0.218 | Viscosity | 0.598 | 0.091 |  |
| Viscosity | 0.598 | 0.081 | Bitterness | 0.934 | 0.529 | Saltiness | 0.396 | 0.224 | Tingling | 0.722 | 0.001 | Bitterness | 0.812 | 0.293 | Saltiness | 0.510 | 0.283 |  |
| Sweetness | 1.141 | 0.478 | Drying | 0.712 | 0.593 | Acidity | 3.052 | 0.244 | Sweetness | 0.917 | < 0.0001 | Tingling | 0.598 | 0.345 | Acidity | 3.046 | 0.343 |  |
| Acidity | 3.019 | 0.672 | Viscosity | 0.678 | 0.644 | Sweetness | 1.125 | 0.313 | Drying | 0.790 | 0.452 | Viscosity | 0.732 | 0.706 | Tingling | 0.489 | 0.349 |  |
| Saltiness | 0.436 | 0.728 | Sweetness | 1.162 | 0.692 | Drying | 0.702 | 0.449 | Bitterness | 0.926 | 0.578 | Sweetness | 1.213 | 0.742 | Drying | 0.798 | 0.409 |  |
| Tingling | 0.534 | 0.874 | Saltiness | 0.473 | 0.712 | Tingling | 0.582 | 0.458 | Viscosity | 0.675 | 0.588 | Saltiness | 0.442 | 0.830 | Bitterness | 0.865 | 0.747 |  |
| **Garganega** | | | **Gewürztraminer** | | | **Greco di Tufo** | | | **Lugana** | | | **Müller Thurgau** | | | **Nosiola** | | |  |
| **Descriptor** | **Estimated mean** | **p-value** | **Descriptor** | **Estimated mean** | **p-value** | **Descriptor** | **Estimated mean** | **p-value** | **Descriptor** | **Estimated mean** | **p-value** | **Descriptor** | **Estimated mean** | **p-value** | **Descriptor** | **Estimated mean** | **p-value** |  |
| Drying | 0.493 | < 0.0001 | Viscosity | 1.272 | < 0.0001 | Acidity | 3.119 | 0.018 | Drying | 0.579 | 0.001 | Bitterness | 0.760 | 0.076 | Acidity | 3.127 | 0.015 |  |
| Bitterness | 0.802 | 0.250 | Sweetness | 2.453 | < 0.0001 | Saltiness | 0.575 | 0.020 | Bitterness | 0.756 | 0.023 | Acidity | 3.074 | 0.137 | Sweetness | 0.934 | 0.001 |  |
| Sweetness | 1.231 | 0.577 | Acidity | 2.532 | < 0.0001 | Drying | 0.835 | 0.160 | Saltiness | 0.510 | 0.178 | Sweetness | 1.099 | 0.202 | Tingling | 0.438 | 0.080 |  |
| Tingling | 0.512 | 0.607 | Saltiness | 0.260 | < 0.0001 | Sweetness | 1.160 | 0.676 | Viscosity | 0.767 | 0.257 | Saltiness | 0.510 | 0.283 | Bitterness | 0.766 | 0.103 |  |
| Viscosity | 0.675 | 0.629 | Drying | 0.281 | < 0.0001 | Tingling | 0.564 | 0.716 | Acidity | 3.033 | 0.402 | Viscosity | 0.748 | 0.532 | Saltiness | 0.526 | 0.181 |  |
| Saltiness | 0.469 | 0.779 | Tingling | 0.365 | 0.001 | Bitterness | 0.864 | 0.731 | Tingling | 0.576 | 0.472 | Tingling | 0.524 | 0.742 | Drying | 0.771 | 0.701 |  |
| Acidity | 3.003 | 0.928 | Bitterness | 0.921 | 0.636 | Viscosity | 0.700 | 0.907 | Sweetness | 1.225 | 0.534 | Drying | 0.760 | 0.824 | Viscosity | 0.720 | 0.851 |  |
| **Pallagrello** | | | **Pinot Grigio** | | | **Ribolla Gialla** | | | **Verdicchio** | | | **Vermentino** | | | **Vernaccia** | | |  |
| **Descriptor** | **Estimated mean** | **p-value** | **Descriptor** | **Estimated mean** | **p-value** | **Descriptor** | **Estimated mean** | **p-value** | **Descriptor** | **Estimated mean** | **p-value** | **Descriptor** | **Estimated mean** | **p-value** | **Descriptor** | **Estimated mean** | **p-value** |  |
| Drying | 1.867 | < 0.0001 | Drying | 0.497 | < 0.0001 | Drying | 0.579 | 0.007 | Drying | 0.546 | 0.009 | Bitterness | 1.076 | 0.008 | Saltiness | 0.344 | 0.096 |  |
| Bitterness | 1.171 | 0.000 | Acidity | 2.882 | 0.026 | Bitterness | 0.821 | 0.337 | Viscosity | 0.620 | 0.258 | Drying | 0.637 | 0.078 | Viscosity | 0.576 | 0.106 |  |
| Tingling | 0.402 | 0.016 | Saltiness | 0.352 | 0.063 | Acidity | 2.958 | 0.406 | Sweetness | 1.097 | 0.273 | Acidity | 2.945 | 0.269 | Sweetness | 1.049 | 0.117 |  |
| Sweetness | 1.026 | 0.021 | Sweetness | 1.135 | 0.459 | Viscosity | 0.662 | 0.466 | Tingling | 0.618 | 0.277 | Sweetness | 1.219 | 0.673 | Drying | 0.654 | 0.252 |  |
| Viscosity | 0.649 | 0.365 | Bitterness | 0.852 | 0.629 | Saltiness | 0.421 | 0.520 | Acidity | 2.968 | 0.603 | Viscosity | 0.732 | 0.699 | Bitterness | 0.958 | 0.446 |  |
| Saltiness | 0.418 | 0.499 | Tingling | 0.521 | 0.711 | Sweetness | 1.168 | 0.751 | Bitterness | 0.868 | 0.811 | Tingling | 0.545 | 0.977 | Tingling | 0.526 | 0.815 |  |
| Acidity | 2.970 | 0.562 | Viscosity | 0.710 | 0.974 | Tingling | 0.555 | 0.830 | Saltiness | 0.444 | 0.884 | Saltiness | 0.454 | 0.995 | Acidity | 2.987 | 0.853 |  |
| Blue: positive model coefficients for descriptors with adjusted means significantly higher than the global mean.  Red: negative model coefficients for descriptors with adjusted means significantly lower than the global mean  Black: not significant. | | | | | | | | | | | | | | | | | |  |

| **Gewürztraminer** | | | | **Vermentino** | | | | **Falanghina** | | | |
| --- | --- | --- | --- | --- | --- | --- | --- | --- | --- | --- | --- |
| **Cluster** | **Descriptor** | **Estimated mean** | **p-value** | **Cluster** | **Descriptor** | **Estimated mean** | **p-value** | **Cluster** | **Descriptor** | **Estimated mean** | **p-value** |
| 3 | Mango | 0.81 | < 0.0001 | 1 | Tropical fruit | 0.76 | < 0.0001 | 1 | Fruity | 1.71 | < 0.0001 |
| 3 | Floral | 1.51 | < 0.0001 | 1 | Aromatic herbs | 0.30 | 0.01 | 1 | Banana | 0.46 | < 0.0001 |
| 3 | Rose | 2.27 | < 0.0001 | 4 | Oxidized | 0.37 | 0.01 | 4 | Sweet odors | 0.75 | < 0.0001 |
| 3 | Orange blossom | 0.38 | < 0.0001 | 4 | Undergrowth | 0.20 | 0.02 | 3 | Floral | 0.97 | 0.03 |
| 3 | Vanilla | 0.46 | < 0.0001 | 4 | Dried fruit | 0.18 | 0.04 | 4 | Off-odors | 0.30 | < 0.0001 |
| 1 | Tropical fruit | 0.70 | 0.04 | 2 | Mineral | 0.43 | 0.01 | 2 | Mineral | 0.37 | < 0.0001 |
| 1 | Fruity | 0.68 | < 0.0001 |  | Off-odors | 0.69 | 0.09 | 2 | Thiolic (S. Blanc profile) | 0.09 | 0.01 |
| 4 | Toasted | 0.02 | < 0.0001 |  | Spicy | 0.39 | 0.11 |  | Smoked | 0.03 | 0.09 |
| 4 | Lactic | 0.06 | < 0.0001 |  | Balsamic | 0.20 | 0.16 |  | Boxtree/Cat pee | 0.05 | 0.10 |
| 2 | Mineral | 0.16 | < 0.0001 |  | Sweet odors | 0.45 | 0.21 |  | Citric | 0.49 | 0.13 |
| 4 | Off-odors | 0.14 | < 0.0001 |  | Smoked | 0.05 | 0.27 |  | Vegetal | 0.20 | 0.16 |
| 4 | Dried fruit | 0.02 | < 0.0001 |  | Vanilla | 0.14 | 0.32 |  | Balsamic | 0.31 | 0.17 |
| 4 | Ethereal/Alcohol | 0.03 | < 0.0001 |  | Toasted | 0.18 | 0.37 |  | Dehydrated fruit | 0.13 | 0.20 |
| 4 | Undergrowth | 0.02 | < 0.0001 |  | Woody | 0.10 | 0.45 |  | Spicy | 0.38 | 0.24 |
| 1 | Citric | 0.39 | < 0.0001 |  | Boxtree/Cat pee | 0.12 | 0.48 |  | Tropical fruit | 0.64 | 0.29 |
| 1 | Balsamic | 0.14 | < 0.0001 |  | Lactic | 0.20 | 0.52 |  | Passion fruit | 0.04 | 0.29 |
| 4 | Smoked | 0.00 | < 0.0001 |  | Grapefruit | 0.17 | 0.58 |  | Undergrowth | 0.09 | 0.30 |
| 4 | Oxidized | 0.10 | < 0.0001 |  | Fruity | 1.18 | 0.73 |  | Dried fruit | 0.15 | 0.33 |
| 4 | Woody | 0.04 | 0.01 |  | Vegetal | 0.25 | 0.75 |  | Orange blossom | 0.21 | 0.36 |
| 2 | Thiolic (S. Blanc profile) | 0.12 | 0.02 |  | Orange blossom | 0.16 | 0.76 |  | Vanilla | 0.23 | 0.37 |
| 2 | Boxtree/Cat pee | 0.04 | 0.03 |  | Ethereal/Alcohol | 0.17 | 0.80 |  | Woody | 0.10 | 0.43 |
| 4 | Dehydrated fruit | 0.04 | 0.04 |  | Dehydrated fruit | 0.10 | 0.82 |  | Oxidized | 0.20 | 0.44 |
|  | Vegetal | 0.18 | 0.05 |  | Passion fruit | 0.07 | 0.84 |  | Mango | 0.27 | 0.44 |
|  | Grapefruit | 0.11 | 0.06 |  | Rose | 0.25 | 0.85 |  | Aromatic herbs | 0.19 | 0.47 |
|  | Spicy | 0.25 | 0.11 |  | Citric | 0.57 | 0.87 |  | Grapefruit | 0.16 | 0.48 |
|  | Banana | 0.19 | 0.23 |  | Banana | 0.25 | 0.90 |  | Rose | 0.20 | 0.74 |
|  | Passion fruit | 0.04 | 0.40 |  | Mango | 0.22 | 0.92 |  | Toasted | 0.16 | 0.75 |
|  | Sweet odors | 0.59 | 0.41 |  | Thiolic (S. Blanc profile) | 0.22 | 0.94 |  | Lactic | 0.23 | 0.76 |
|  | Aromatic herbs | 0.21 | 0.80 |  | Floral | 0.72 | 0.97 |  | Ethereal/Alcohol | 0.17 | 0.80 |
| Blue: positive model coefficients for descriptors with adjusted means significantly higher than the global mean. | | | | | | | |  |  |  |  |
| Red: negative model coefficients for descriptors with adjusted means significantly lower than the global mean. | | | | | | | |  |  |  |  |
| Black: not significant. | |  |  |  |  |  |  |  |  |  |  |

**Table S3.** Descriptive and discriminative olfactory characteristics specific to each monovarietal wine. Descriptor, Estimated mean, p-value for each combination product-olfactory descriptor are reported. The column "Cluster" refers to the HCA in Figure 2.

**Table S3. Continued**

| **Greco di Tufo** | | | | **Garganega** | | | | **Verdicchio** | | | |
| --- | --- | --- | --- | --- | --- | --- | --- | --- | --- | --- | --- |
| **Cluster** | **Descriptor** | **Estimated mean** | **p-value** | **Cluster** | **Descriptor** | **Estimated mean** | **p-value** | **Cluster** | **Descriptor** | **Estimated mean** | **p-value** |
| 1 | Banana | 0.41 | < 0.0001 | 1 | Citric | 0.75 | 0.01 | 1 | Fruity | 1.49 | 0.01 |
| 1 | Fruity | 1.48 | < 0.0001 | 2 | Boxtree/Cat pee | 0.16 | 0.02 | 2 | Mineral | 0.78 | 0.01 |
| 3 | Vanilla | 0.29 | 0.03 | 2 | Mineral | 0.72 | 0.04 | 4 | Spicy | 0.47 | 0.01 |
| 4 | Sweet odors | 0.69 | 0.03 | 1 | Fruity | 1.39 | 0.04 | 1 | Citric | 0.77 | 0.02 |
| 4 | Oxidized | 0.13 | 0.03 |  | Oxidized | 0.15 | 0.07 | 4 | Off-odors | 0.34 | < 0.0001 |
| 2 | Mineral | 0.46 | 0.03 |  | Dehydrated fruit | 0.05 | 0.12 | 4 | Oxidized | 0.10 | 0.02 |
| 4 | Off-odors | 0.44 | 0.04 |  | Rose | 0.08 | 0.13 |  | Balsamic | 0.33 | 0.12 |
|  | Rose | 0.07 | 0.08 |  | Vegetal | 0.33 | 0.21 |  | Aromatic herbs | 0.28 | 0.12 |
|  | Thiolic (S. Blanc profile) | 0.15 | 0.13 |  | Undergrowth | 0.09 | 0.22 |  | Toasted | 0.21 | 0.16 |
|  | Boxtree/Cat pee | 0.06 | 0.17 |  | Balsamic | 0.21 | 0.27 |  | Undergrowth | 0.09 | 0.31 |
|  | Spicy | 0.26 | 0.18 |  | Smoked | 0.11 | 0.31 |  | Tropical fruit | 0.48 | 0.32 |
|  | Undergrowth | 0.09 | 0.22 |  | Floral | 0.59 | 0.32 |  | Passion fruit | 0.03 | 0.36 |
|  | Balsamic | 0.30 | 0.23 |  | Orange blossom | 0.12 | 0.32 |  | Vegetal | 0.22 | 0.37 |
|  | Citric | 0.66 | 0.24 |  | Dried fruit | 0.10 | 0.33 |  | Orange blossom | 0.22 | 0.40 |
|  | Passion fruit | 0.03 | 0.26 |  | Spicy | 0.28 | 0.46 |  | Rose | 0.15 | 0.49 |
|  | Smoked | 0.05 | 0.29 |  | Toasted | 0.13 | 0.48 |  | Sweet odors | 0.48 | 0.50 |
|  | Dehydrated fruit | 0.12 | 0.36 |  | Off-odors | 0.53 | 0.51 |  | Vanilla | 0.15 | 0.55 |
|  | Aromatic herbs | 0.19 | 0.39 |  | Ethereal/Alcohol | 0.14 | 0.64 |  | Floral | 0.78 | 0.59 |
|  | Tropical fruit | 0.50 | 0.41 |  | Mango | 0.19 | 0.67 |  | Mango | 0.25 | 0.66 |
|  | Ethereal/Alcohol | 0.13 | 0.49 |  | Tropical fruit | 0.53 | 0.68 |  | Thiolic (S. Blanc profile) | 0.20 | 0.66 |
|  | Vegetal | 0.25 | 0.63 |  | Passion fruit | 0.06 | 0.80 |  | Dehydrated fruit | 0.08 | 0.68 |
|  | Floral | 0.67 | 0.74 |  | Woody | 0.12 | 0.80 |  | Woody | 0.11 | 0.69 |
|  | Grapefruit | 0.18 | 0.80 |  | Thiolic (S. Blanc profile) | 0.23 | 0.85 |  | Grapefruit | 0.21 | 0.72 |
|  | Lactic | 0.23 | 0.86 |  | Lactic | 0.23 | 0.87 |  | Dried fruit | 0.11 | 0.77 |
|  | Woody | 0.12 | 0.88 |  | Sweet odors | 0.55 | 0.88 |  | Ethereal/Alcohol | 0.15 | 0.81 |
|  | Mango | 0.22 | 0.89 |  | Vanilla | 0.19 | 0.90 |  | Boxtree/Cat pee | 0.10 | 0.87 |
|  | Toasted | 0.15 | 0.97 |  | Banana | 0.25 | 0.91 |  | Lactic | 0.22 | 0.92 |
|  | Dried fruit | 0.12 | 0.98 |  | Grapefruit | 0.19 | 0.98 |  | Smoked | 0.08 | 0.95 |
|  | Orange blossom | 0.17 | 1.00 |  | Aromatic herbs | 0.22 | 0.98 |  | Banana | 0.24 | 0.95 |
| Blue: positive model coefficients for descriptors with adjusted means significantly higher than the global mean. | | | | | | | |  |  |  |  |
| Red: negative model coefficients for descriptors with adjusted means significantly lower than the global mean. | | | | | | | |  |  |  |  |
| Black: not significant. | |  |  |  |  |  |  |  |  |  |  |

**Table S3. Continued**

| **Pinot Grigio** | | | | **Lugana** | | | | **Müller Thurgau** | | | |
| --- | --- | --- | --- | --- | --- | --- | --- | --- | --- | --- | --- |
| **Cluster** | **Descriptor** | **Estimated mean** | **p-value** | **Cluster** | **Descriptor** | **Estimated mean** | **p-value** | **Cluster** | **Descriptor** | **Estimated mean** | **p-value** |
| 4 | Off-odors (Sulphureous) | 0.84 | < 0.0001 | 2 | Thiolic (S. Blanc profile) | 0.35 | < 0.0001 | 2 | Passion fruit | 0.34 | < 0.0001 |
| 2 | Mineral | 0.76 | 0.01 | 1 | Aromatic herbs | 0.30 | < 0.0001 | 2 | Thiolic (S. Blanc profile) | 0.57 | < 0.0001 |
| 1 | Citric | 0.74 | 0.02 | 2 | Vegetal | 0.38 | < 0.0001 | 2 | Boxtree/Cat pee | 0.32 | < 0.0001 |
| 4 | Sweet odors | 0.35 | 0.01 | 2 | Mineral | 0.74 | < 0.0001 | 1 | Tropical fruit | 0.79 | < 0.0001 |
| 3 | Floral | 0.41 | 0.01 | 1 | Citric | 0.68 | 0.05 | 2 | Grapefruit | 0.34 | < 0.0001 |
| 1 | Aromatic herbs | 0.15 | 0.05 | 4 | Sweet odors | 0.38 | 0.01 | 1 | Aromatic herbs | 0.31 | < 0.0001 |
|  | Lactic | 0.29 | 0.07 |  | Rose | 0.09 | 0.07 | 2 | Vegetal | 0.39 | 0.01 |
|  | Smoked | 0.13 | 0.10 |  | Orange blossom | 0.11 | 0.11 | 2 | Mineral | 0.74 | 0.02 |
|  | Spicy | 0.25 | 0.13 |  | Dehydrated fruit | 0.07 | 0.21 | 4 | Sweet odors | 0.35 | 0.01 |
|  | Vegetal | 0.34 | 0.13 |  | Balsamic | 0.22 | 0.29 | 4 | Oxidized | 0.10 | 0.01 |
|  | Rose | 0.10 | 0.18 |  | Floral | 0.62 | 0.31 | 4 | Ethereal/Alcohol | 0.08 | 0.03 |
|  | Passion fruit | 0.03 | 0.20 |  | Passion fruit | 0.09 | 0.35 | 4 | Dried fruit | 0.06 | 0.03 |
|  | Dehydrated fruit | 0.14 | 0.21 |  | Mango | 0.17 | 0.40 |  | Balsamic | 0.18 | 0.06 |
|  | Grapefruit | 0.13 | 0.22 |  | Oxidized | 0.28 | 0.45 |  | Toasted | 0.09 | 0.07 |
|  | Toasted | 0.19 | 0.23 |  | Smoked | 0.10 | 0.45 |  | Citric | 0.70 | 0.07 |
|  | Thiolic (S. Blanc profile) | 0.28 | 0.24 |  | Grapefruit | 0.22 | 0.52 |  | Vanilla | 0.11 | 0.08 |
|  | Ethereal/Alcohol | 0.11 | 0.25 |  | Tropical fruit | 0.59 | 0.60 |  | Dehydrated fruit | 0.04 | 0.09 |
|  | Fruity | 1.11 | 0.27 |  | Vanilla | 0.20 | 0.61 |  | Lactic | 0.17 | 0.16 |
|  | Balsamic | 0.22 | 0.33 |  | Banana | 0.26 | 0.63 |  | Banana | 0.19 | 0.25 |
|  | Mango | 0.16 | 0.39 |  | Dried fruit | 0.11 | 0.66 |  | Woody | 0.09 | 0.27 |
|  | Orange blossom | 0.14 | 0.49 |  | Lactic | 0.23 | 0.68 |  | Floral | 0.60 | 0.33 |
|  | Boxtree/Cat pee | 0.11 | 0.54 |  | Spicy | 0.34 | 0.69 |  | Smoked | 0.05 | 0.38 |
|  | Undergrowth | 0.11 | 0.56 |  | Woody | 0.12 | 0.71 |  | Fruity | 1.14 | 0.39 |
|  | Dried fruit | 0.14 | 0.61 |  | Undergrowth | 0.12 | 0.72 |  | Undergrowth | 0.16 | 0.40 |
|  | Tropical fruit | 0.54 | 0.77 |  | Toasted | 0.14 | 0.74 |  | Orange blossom | 0.20 | 0.57 |
|  | Vanilla | 0.18 | 0.87 |  | Boxtree/Cat pee | 0.10 | 0.86 |  | Off-odors | 0.60 | 0.73 |
|  | Oxidized | 0.24 | 0.90 |  | Ethereal/Alcohol | 0.15 | 0.88 |  | Spicy | 0.33 | 0.77 |
|  | Woody | 0.12 | 0.91 |  | Fruity | 1.22 | 0.92 |  | Rose | 0.21 | 0.84 |
|  | Banana | 0.24 | 0.96 |  | Off-odors | 0.58 | 1.00 |  | Mango | 0.21 | 0.95 |
| Blue: positive model coefficients for descriptors with adjusted means significantly higher than the global mean. | | | | | | | |  |  |  |  |
| Red: negative model coefficients for descriptors with adjusted means significantly lower than the global mean. | | | | | | | |  |  |  |  |
| Black: not significant. | |  |  |  |  |  |  |  |  |  |  |
|  |  |  |  |  |  |  |  |  |  |  |  |

**Table S3. Continued**

| **Albana** | | | | **Pallagrello** | | | | **Erbaluce** | | | |
| --- | --- | --- | --- | --- | --- | --- | --- | --- | --- | --- | --- |
| **Cluster** | **Descriptor** | **Estimated mean** | **p-value** | **Cluster** | **Descriptor** | **Estimated mean** | **p-value** | **Cluster** | **Descriptor** | **Estimated mean** | **p-value** |
| 4 | Off-odors | 0.97 | < 0.0001 | 4 | Off-odors | 1.07 | < 0.0001 | 4 | Off-odors | 0.74 | 0.01 |
| 4 | Ethereal/Alcohol | 0.30 | < 0.0001 | 4 | Oxidized | 0.43 | < 0.0001 | 4 | Undergrowth | 0.19 | 0.04 |
| 4 | Toasted | 0.27 | < 0.0001 | 4 | Undergrowth | 0.24 | < 0.0001 | 1 | Banana | 0.12 | 0.01 |
| 4 | Lactic | 0.35 | < 0.0001 | 4 | Ethereal/Alcohol | 0.26 | 0.01 | 3 | Rose | 0.04 | 0.03 |
| 4 | Smoked | 0.16 | < 0.0001 | 1 | Fruity | 0.92 | < 0.0001 | 4 | Toasted | 0.09 | 0.04 |
| 4 | Oxidized | 0.40 | < 0.0001 | 1 | Tropical fruit | 0.38 | 0.01 | 4 | Dried fruit | 0.07 | 0.05 |
| 4 | Dehydrated fruit | 0.18 | < 0.0001 | 3 | Orange blossom | 0.07 | 0.03 |  | Vanilla | 0.11 | 0.06 |
| 4 | Spicy | 0.45 | 0.01 | 3 | Floral | 0.48 | 0.04 |  | Spicy | 0.24 | 0.07 |
| 4 | Sweet odors | 0.70 | 0.02 | 3 | Rose | 0.04 | 0.04 |  | Balsamic | 0.32 | 0.07 |
| 4 | Dried fruit | 0.19 | 0.02 | 3 | Mango | 0.08 | 0.05 |  | Mango | 0.11 | 0.09 |
| 4 | Woody | 0.19 | 0.04 |  | Vanilla | 0.11 | 0.08 |  | Grapefruit | 0.26 | 0.14 |
| 1 | Aromatic herbs | 0.12 | < 0.0001 |  | Grapefruit | 0.13 | 0.15 |  | Woody | 0.08 | 0.20 |
| 1 | Citric | 0.42 | 0.01 |  | Toasted | 0.10 | 0.17 |  | Mineral | 0.66 | 0.20 |
| 1 | Balsamic | 0.16 | 0.01 |  | Dehydrated fruit | 0.06 | 0.22 |  | Oxidized | 0.30 | 0.30 |
| 2 | Boxtree/Cat pee | 0.03 | 0.02 |  | Mineral | 0.66 | 0.23 |  | Citric | 0.64 | 0.31 |
| 3 | Rose | 0.04 | 0.04 |  | Balsamic | 0.30 | 0.24 |  | Vegetal | 0.22 | 0.32 |
| 2 | Vegetal | 0.18 | 0.05 |  | Boxtree/Cat pee | 0.06 | 0.25 |  | Smoked | 0.10 | 0.32 |
|  | Tropical fruit | 0.43 | 0.06 |  | Banana | 0.29 | 0.30 |  | Lactic | 0.19 | 0.34 |
|  | Fruity | 1.07 | 0.09 |  | Citric | 0.52 | 0.32 |  | Thiolic (S. Blanc profile) | 0.18 | 0.42 |
|  | Passion fruit | 0.02 | 0.11 |  | Passion fruit | 0.04 | 0.44 |  | Floral | 0.64 | 0.52 |
|  | Grapefruit | 0.13 | 0.13 |  | Dried fruit | 0.14 | 0.46 |  | Fruity | 1.16 | 0.54 |
|  | Banana | 0.18 | 0.15 |  | Smoked | 0.10 | 0.48 |  | Passion fruit | 0.05 | 0.61 |
|  | Floral | 0.56 | 0.17 |  | Vegetal | 0.24 | 0.56 |  | Ethereal/Alcohol | 0.14 | 0.70 |
|  | Thiolic (S. Blanc profile) | 0.16 | 0.21 |  | Thiolic (S. Blanc profile) | 0.19 | 0.57 |  | Aromatic herbs | 0.20 | 0.70 |
|  | Undergrowth | 0.15 | 0.54 |  | Woody | 0.14 | 0.72 |  | Dehydrated fruit | 0.10 | 0.77 |
|  | Mango | 0.18 | 0.61 |  | Spicy | 0.30 | 0.72 |  | Boxtree/Cat pee | 0.09 | 0.87 |
|  | Vanilla | 0.18 | 0.83 |  | Aromatic herbs | 0.21 | 0.85 |  | Tropical fruit | 0.55 | 0.88 |
|  | Orange blossom | 0.17 | 1.00 |  | Sweet odors | 0.53 | 0.89 |  | Sweet odors | 0.53 | 0.92 |
|  | Mineral | 0.59 | 1.00 |  | Lactic | 0.22 | 0.97 |  | Orange blossom | 0.17 | 0.99 |
| Blue: positive model coefficients for descriptors with adjusted means significantly higher than the global mean. | | | | | | | |  |  |  |  |
| Red: negative model coefficients for descriptors with adjusted means significantly lower than the global mean. | | | | | | | |  |  |  |  |
| Black: not significant. | |  |  |  |  |  |  |  |  |  |  |

| **Nosiola** | | | | **Arneis** | | | | **Ribolla Gialla** | | | |
| --- | --- | --- | --- | --- | --- | --- | --- | --- | --- | --- | --- |
| **Cluster** | **Descriptor** | **Estimated mean** | **p-value** | **Cluster** | **Descriptor** | **Estimated mean** | **p-value** | **Cluster** | **Descriptor** | **Estimated mean** | **p-value** |
| 4 | Woody | 0.32 | < 0.0001 | 4 | Undergrowth | 0.21 | 0.01 | 4 | Dehydrated fruit | 0.18 | 0.01 |
| 4 | Ethereal/Alcohol | 0.27 | < 0.0001 | 4 | Lactic | 0.30 | 0.04 | 1 | Aromatic herbs | 0.13 | 0.01 |
| 4 | Oxidized | 0.42 | < 0.0001 | 1 | Tropical fruit | 0.37 | 0.01 | 4 | Off-odors | 0.42 | 0.02 |
| 4 | Toasted | 0.23 | 0.01 | 1 | Fruity | 0.99 | 0.01 | 2 | Vegetal | 0.17 | 0.03 |
| 2 | Boxtree/Cat pee | 0.16 | 0.04 | 3 | Floral | 0.43 | 0.02 | 2 | Boxtree/Cat pee | 0.04 | 0.04 |
| 1 | Fruity | 1.01 | 0.02 | 1 | Citric | 0.44 | 0.02 | 1 | Balsamic | 0.18 | 0.05 |
| 1 | Banana | 0.14 | 0.04 | 3 | Rose | 0.02 | 0.02 |  | Toasted | 0.21 | 0.06 |
|  | Citric | 0.47 | 0.07 | 3 | Mango | 0.08 | 0.04 |  | Citric | 0.48 | 0.09 |
|  | Rose | 0.10 | 0.19 |  | Toasted | 0.21 | 0.06 |  | Thiolic (S. Blanc profile) | 0.30 | 0.09 |
|  | Orange blossom | 0.11 | 0.24 |  | Smoked | 0.13 | 0.06 |  | Fruity | 1.33 | 0.14 |
|  | Off-odors | 0.66 | 0.29 |  | Off-odors | 0.71 | 0.07 |  | Rose | 0.11 | 0.17 |
|  | Tropical fruit | 0.49 | 0.32 |  | Grapefruit | 0.28 | 0.08 |  | Lactic | 0.17 | 0.21 |
|  | Mango | 0.15 | 0.35 |  | Oxidized | 0.33 | 0.12 |  | Passion fruit | 0.10 | 0.21 |
|  | Spicy | 0.37 | 0.36 |  | Banana | 0.17 | 0.16 |  | Spicy | 0.38 | 0.23 |
|  | Undergrowth | 0.15 | 0.50 |  | Balsamic | 0.21 | 0.19 |  | Orange blossom | 0.12 | 0.25 |
|  | Sweet odors | 0.58 | 0.58 |  | Woody | 0.17 | 0.19 |  | Woody | 0.16 | 0.28 |
|  | Passion fruit | 0.08 | 0.60 |  | Mineral | 0.67 | 0.19 |  | Dried fruit | 0.15 | 0.29 |
|  | Dehydrated fruit | 0.08 | 0.64 |  | Passion fruit | 0.03 | 0.22 |  | Tropical fruit | 0.63 | 0.36 |
|  | Balsamic | 0.24 | 0.69 |  | Dehydrated fruit | 0.06 | 0.22 |  | Oxidized | 0.29 | 0.37 |
|  | Dried fruit | 0.13 | 0.70 |  | Sweet odors | 0.47 | 0.31 |  | Vanilla | 0.15 | 0.41 |
|  | Thiolic (S. Blanc profile) | 0.20 | 0.71 |  | Aromatic herbs | 0.18 | 0.31 |  | Mango | 0.17 | 0.43 |
|  | Vegetal | 0.25 | 0.72 |  | Spicy | 0.28 | 0.39 |  | Mineral | 0.55 | 0.50 |
|  | Vanilla | 0.17 | 0.75 |  | Vanilla | 0.15 | 0.49 |  | Banana | 0.27 | 0.57 |
|  | Mineral | 0.57 | 0.76 |  | Orange blossom | 0.14 | 0.50 |  | Sweet odors | 0.51 | 0.65 |
|  | Aromatic herbs | 0.22 | 0.86 |  | Thiolic (S. Blanc profile) | 0.19 | 0.54 |  | Floral | 0.74 | 0.81 |
|  | Lactic | 0.21 | 0.88 |  | Dried fruit | 0.13 | 0.71 |  | Smoked | 0.07 | 0.83 |
|  | Smoked | 0.07 | 0.89 |  | Boxtree/Cat pee | 0.09 | 0.74 |  | Undergrowth | 0.13 | 0.92 |
|  | Grapefruit | 0.20 | 0.92 |  | Vegetal | 0.26 | 0.90 |  | Grapefruit | 0.19 | 0.93 |
|  | Floral | 0.71 | 0.99 |  | Ethereal/Alcohol | 0.16 | 0.94 |  | Ethereal/Alcohol | 0.16 | 0.96 |
| Blue: positive model coefficients for descriptors with adjusted means significantly higher than the global mean. | | | | | | | |  |  |  |  |
| Red: negative model coefficients for descriptors with adjusted means significantly lower than the global mean. | | | | | | | |  |  |  |  |
| Black: not significant. | |  |  |  |  |  |  |  |  |  |  |

**Table S3. Continued**

**Table S3. Continued**

| **Cortese** | | | | **Vernaccia** | | | | **Fiano** | | | |
| --- | --- | --- | --- | --- | --- | --- | --- | --- | --- | --- | --- |
| **Cluster** | **Descriptor** | **Estimated mean** | **p-value** | **Cluster** | **Descriptor** | **Estimated mean** | **p-value** | **Cluster** | **Descriptor** | **Estimated mean** | **p-value** |
| 1 | Balsamic | 0.39 | < 0.0001 | 1 | Balsamic | 0.43 | < 0.0001 | 3 | Passion fruit | 0.00 | 0.02 |
| 1 | Banana | 0.37 | 0.01 |  | Banana | 0.13 | 0.06 |  | Undergrowth | 0.07 | 0.09 |
| 4 | Oxidized | 0.11 | 0.01 |  | Vanilla | 0.08 | 0.07 |  | Smoked | 0.03 | 0.09 |
| 4 | Off-odors | 0.46 | 0.05 |  | Spicy | 0.22 | 0.10 |  | Off-odors | 0.47 | 0.10 |
|  | Woody | 0.08 | 0.12 |  | Sweet odors | 0.67 | 0.14 |  | Dried fruit | 0.17 | 0.12 |
|  | Dehydrated fruit | 0.05 | 0.13 |  | Toasted | 0.09 | 0.16 |  | Spicy | 0.25 | 0.13 |
|  | Ethereal/Alcohol | 0.11 | 0.16 |  | Boxtree/Cat pee | 0.05 | 0.23 |  | Rose | 0.11 | 0.19 |
|  | Dried fruit | 0.09 | 0.18 |  | Mineral | 0.68 | 0.26 |  | Floral | 0.85 | 0.21 |
|  | Toasted | 0.11 | 0.20 |  | Off-odors | 0.48 | 0.27 |  | Vegetal | 0.32 | 0.24 |
|  | Rose | 0.14 | 0.31 |  | Fruity | 1.32 | 0.28 |  | Grapefruit | 0.25 | 0.26 |
|  | Sweet odors | 0.60 | 0.33 |  | Woody | 0.08 | 0.31 |  | Mineral | 0.52 | 0.28 |
|  | Fruity | 1.28 | 0.33 |  | Vegetal | 0.33 | 0.32 |  | Citric | 0.65 | 0.28 |
|  | Mango | 0.16 | 0.36 |  | Rose | 0.13 | 0.37 |  | Fruity | 1.30 | 0.29 |
|  | Vanilla | 0.22 | 0.38 |  | Dehydrated fruit | 0.13 | 0.40 |  | Toasted | 0.12 | 0.30 |
|  | Vegetal | 0.30 | 0.39 |  | Grapefruit | 0.15 | 0.42 |  | Thiolic (S. Blanc profile) | 0.17 | 0.31 |
|  | Tropical fruit | 0.62 | 0.40 |  | Citric | 0.52 | 0.44 |  | Lactic | 0.26 | 0.33 |
|  | Undergrowth | 0.10 | 0.41 |  | Undergrowth | 0.10 | 0.45 |  | Vanilla | 0.22 | 0.43 |
|  | Orange blossom | 0.20 | 0.43 |  | Thiolic (S. Blanc profile) | 0.18 | 0.55 |  | Boxtree/Cat pee | 0.07 | 0.44 |
|  | Boxtree/Cat pee | 0.08 | 0.44 |  | Lactic | 0.19 | 0.57 |  | Mango | 0.25 | 0.65 |
|  | Spicy | 0.35 | 0.50 |  | Dried fruit | 0.14 | 0.60 |  | Sweet odors | 0.51 | 0.68 |
|  | Citric | 0.62 | 0.51 |  | Orange blossom | 0.19 | 0.69 |  | Dehydrated fruit | 0.11 | 0.70 |
|  | Aromatic herbs | 0.23 | 0.54 |  | Floral | 0.77 | 0.69 |  | Ethereal/Alcohol | 0.14 | 0.71 |
|  | Mineral | 0.56 | 0.62 |  | Oxidized | 0.22 | 0.73 |  | Orange blossom | 0.18 | 0.83 |
|  | Floral | 0.75 | 0.73 |  | Passion fruit | 0.05 | 0.77 |  | Balsamic | 0.25 | 0.89 |
|  | Passion fruit | 0.06 | 0.81 |  | Mango | 0.19 | 0.79 |  | Woody | 0.12 | 0.92 |
|  | Thiolic (S. Blanc profile) | 0.21 | 0.82 |  | Smoked | 0.07 | 0.81 |  | Oxidized | 0.24 | 0.94 |
|  | Lactic | 0.23 | 0.87 |  | Aromatic herbs | 0.22 | 0.85 |  | Banana | 0.24 | 0.94 |
|  | Grapefruit | 0.20 | 0.96 |  | Ethereal/Alcohol | 0.15 | 0.86 |  | Tropical fruit | 0.56 | 0.97 |
|  | Smoked | 0.08 | 0.97 |  | Tropical fruit | 0.56 | 0.99 |  | Aromatic herbs | 0.22 | 0.99 |
| Blue: positive model coefficients for descriptors with adjusted means significantly higher than the global mean. | | | | | | | |  |  |  |  |
| Red: negative model coefficients for descriptors with adjusted means significantly lower than the global mean. | | | | | | | |  |  |  |  |
| Black: not significant. | |  |  |  |  |  |  |  |  |  |  |
